# Extending the PARCH Scale: Assessing Hydropathy of Proteins across Multiple Water Models

**DOI:** 10.1021/acs.jcim.4c02415

**Published:** 2025-03-04

**Authors:** Xuyang Qin, Jingjing Ji, Somya Chakraborty, Shikha Nangia

**Affiliations:** Department of Biomedical and Chemical Engineering, Syracuse University, Syracuse, New York 13244, United States

## Abstract

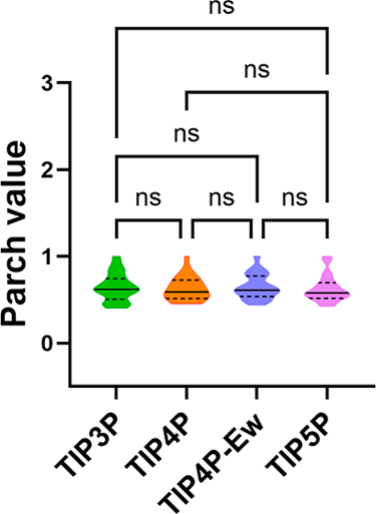

Quantitative assessment of amino acid hydropathy can be done using the protocol for assigning a residue’s character on a hydropathy (PARCH) scale, which assigns values from 0 to 10, with lower values indicating greater hydrophobicity. The merit of the PARCH scale lies in its ability to integrate both the nanoscale topographical features and the chemical properties of amino acid residues when determining hydropathy. In its initial application, we employed the TIP3P water model, optimized for CHARMM36m proteins, to simulate the water behavior around the protein surface. Due to the growing use of the PARCH scale, we have extended its application to three additional all-atom water models: TIP4P, TIP4P-Ew, and TIP5P. Our findings reveal that although PARCH values vary across these water models, the relative hydropathy trends remain consistent. All models successfully distinguished hydrophobic from hydrophilic regions in nanoscale topography, although charged residues showed greater sensitivity to model choice, leading to more significant value variances. Additionally, we evaluated the influence of two other parameters—the force constant used to constrain proteins and the time step of the evaporation process—on the PARCH scale. Overall, the PARCH scale has demonstrated robustness in capturing protein hydropathy across various water models, suggesting its potential applicability with other protein–water force field combinations and even molecular systems beyond proteins.

## 1 Introduction

Hydropathy plays a crucial role in biomolecules by determining their complex structures and functions within biological systems. In proteins, it has been well established that matching hydrophobicity and hydrophilicity between amino acid residues drives the folding, stabilizing, and interacting of proteins to fulfill their biological functions.^1−5^ Meanwhile, protein hydropathy is integral to unraveling biological processes like membrane insertion, protein assembly, signal transduction, and enzymatic activity.^5−8^

The chemical characteristics of a side chain can tell whether a single amino acid is hydrophobic or hydrophilic in isolation. However, the hydropathy of amino acid residues on proteins is hard to scale to a fixed value as even slight variances in the side-chain topology or local geometry yield substantial differences in their affinity to water. The nanoscale topography and chemical properties of the protein surface collectively drive the interactions with the aqueous environment.^2^

For decades, no hydropathy scale accounted for both surface topography and chemical properties—until the development of our Protocol for Assigning a Residue’s Character on a Hydropathy (PARCH) scale. The PARCH scale quantifies the ability of amino acids to retain or lose bound water molecules as a function of temperature during a simulated annealing process within a standard molecular dynamics simulation. This straightforward and computationally efficient approach assigns each residue a PARCH value on a scale from 0 to 10: 0 represents a highly hydrophobic amino acid that rapidly loses water or has no access to water, while 10 denotes a highly hydrophilic residue with strong water retention. Our recent work introduced an extensive protein database containing PARCH values of 1000 experimentally determined protein structures, encompassing over 270,000 amino acid residues.^9,10^ This database, encompassing proteins from multiple species, diverse biological functions, and intricate surface topographies, serves as a valuable resource for advancing our understanding of protein assembly. It also provides critical insights for drug delivery and the design of novel proteins with tailored properties and functions.

A critical factor in the PARCH scale’s ability to accurately quantify the hydropathy of a residue is the collective response of the surrounding water molecules. However, different water models may produce inconsistent assessments of protein hydropathy due to variations in their inherent parameterizations. To address this, we set out to evaluate the parallel to PARCHvalues of proteins using well-established water models in biological simulations. With a long-term goal of extending the PARCH scale to other biomolecules—such as nucleic acids, carbohydrates, and lipids—while incorporating various force fields and water models, this study serves as a foundational step in assessing the accuracy of different water models for quantifying protein hydropathy using the PARCH approach.

Various water models have been developed to produce more accurate aqueous properties, align with different force fields, and cater to the specific needs of researchers. TIP3P, TIP4P, TIP4P-Ew, and TIP5P are widely used water models in molecular dynamics simulations (Figure S1), designed to represent water molecules with varying levels of complexity.^15^

TIP3P and TIP4P models were established by Jorgensen and colleagues in the 1980s^11^ and have been widely used and modified into different versions up to the present day (Figure S1). TIP3P is the simplest model and uses three interaction sites—one for the oxygen atom and two for hydrogens. It is computationally efficient and commonly applied in biomolecular simulations, although it has limitations in accurately representing water’s physical properties, such as its dipole moment. Besides the original TIP3P, there exists a modified version tailored for CHARMM36m proteins^12^ showing good production of first-shell hydration and water energetics while keeping relatively low computational cost, which we applied for the original PARCH. TIP4P is a four-point water model whose negative charge is distributed on an additional site along the bisector of the H–O–H bond rather than on oxygen. The fourth site improves water’s dipole moment and the accuracy in reproducing density and melting points.^11^ The parameters are tuned to match liquid water’s experimental density and vaporization enthalpy at 25 °C.

TIP4P-Ew is a reparameterized TIP4P optimized for using the Ewald electrostatics method. It is reported to reproduce experimental bulk densities and the enthalpy of vaporization across a wider temperature range.^13^ TIP5P is a relatively young five-point model, which positions negative charges on two extra sites representing ″lone pair″ electrons. It is reported to exhibit more accurate density across a broad temperature range at 1 atm, and it reproduces the density maximum near 4 °C at 1 atm.^14^ The force field parameters of water models are included in Table 1.

**Table 1:** Partial Charges, Bond Distances, Bond Angles, and Lennard-Jones Parameters for TIP3P, TIP4P, TIP4P-Ew, and TIP5P Water Models^a^

parameters	TIP3P^11,12^	TIP4P^11^	TIP4P-Ew^13^	TIP5P^14^
*q*_H_ (e)	0.417	0.52	0.52422	0.241
*q*_O_ (e)	–0.834	0	0	0
*q*_V_ (e)	-	–1.040	–1.04844	–0.241
*d*_OH_ (nm)	0.09572	0.09572	0.09572	0.09572
*d*_OV_ (nm)	-	0.015	0.0125	0.070
θ_HOH_ (°)	104.52	104.52	104.52	104.52
θ_VOV_ (°)	-	-	-	109.47
σ_O_ (nm)	0.315057	0.315365	0.315365	0.312000
ε_O_ (kJ mol^–1^)	0.63639	0.64852	0.64852	0.66944

a *q*: partial charges for hydrogen (H), oxygen (O), and virtual (V) sites; *d*: distances for oxygen–hydrogen (OH) and oxygen–virtual (OV) sites; θ: angles for hydrogen–oxygen–hydrogen (HOH) and virtual–oxygen–virtual (VOV) sites; σ and ε: Lennard-Jones interaction parameters for oxygen (O).

Each of these water models serves distinct purposes, making them valuable for different types of simulations.^15^ Although the CHARMM-modified TIP3P water model remains the most widely used option for CHARMM36m protein simulations,^16^ TIP4P and TIP5P are reportedly compatible with CHARMM proteins,^17,18^ albeit with mixed opinions regarding their performance.^19−21^ The choice of water model ultimately depends on achieving a balance between computational efficiency and simulation accuracy.^22^ TIP3P is well-suited for general-purpose simulations due to its simplicity and low computational cost.^19,22^ In contrast, TIP4P and TIP4P-Ew are preferred for studies requiring a more accurate representation of water behavior, particularly in condensed-phase systems.^23^ TIP5P is ideal for applications that demand highly detailed modeling of hydrogen bonding and water structuring.^24^

In this study, we investigated the impact of three water force fields—TIP4P, TIP4P-Ew, and TIP5P—on the PARCH values of protein residues and compared these results to previously published data using the TIP3P model.^9^ The objective is to determine whether different water models affect the PARCH values of proteins and if they differentially treat amino acid residues with distinct chemical properties, such as charged versus uncharged, polar versus nonpolar, or aliphatic versus aromatic, due to variations in the water model’s polarity. Rather than seeking the ‘best’ water model for PARCH, we aim to offer a range of potential options and insights to users with preferences for specific water models.

## 2 Approach

The PARCH scale quantifies a residue’s tendency to retain or lose hydration shell water molecules as a function of temperature. To compute PARCH values, the protein is solvated within a uniformly dense water shell, allowing water molecules to conform closely to the protein’s surface and form a nanoscale hydration network around the residues. A nonequilibrium annealing simulation is then conducted, gradually increasing the temperature to evaporate water molecules from the protein’s surface. Throughout this process, the protein is position-restrained to prevent temperature-induced conformational changes in the residues.

The retention or loss of water from a residue is influenced by several factors, including its chemical identity, local nanoscale topography, and chemical properties of neighboring residues. The initial count of water molecules surrounding a residue provides insight into its topographical features, indicating its accessibility to water. During the annealing process, the chemical identity of the residue and its neighbors dictates the nature of their interactions with water, which, in turn, influences the number of water molecules retained at each temperature. For example, residues that interact weakly with water will lose more water molecules than those with stronger water interactions.

A critical aspect of the PARCH scale calculation is the accurate measurement of water molecule counts around each residue while preserving the local nanoscale topography of the residue. To achieve this, the protein’s structure must remain position-restrained throughout the annealing process. Restraining the protein prevents structural fluctuations, denaturation, or loss of its native conformation, ensuring that water interactions are assessed under biologically relevant conditions. Consequently, during annealing, only the temperature of the water molecules is increased, while the protein structure remains fixed.

To compute the PARCH values, we follow the procedure outlined below:

1. Solvation and annealing

- The equilibrated protein structure is solvated with a shell of water of radius *d*_shell_.
- While keeping the protein position restrained, annealing is performed on the water at a fixed rate (gradually increasing temperature) under constant volume conditions.

2. Water molecule count calculation

- At regular intervals during the annealing simulations, the number of water molecules surrounding each residue within a specified cutoff distance, *d*_water_, is computed.
- *w*_*i*_(*t*) is defined as the number of water molecules surrounding the *i*th residue at time *t*.

3. Monotonicity condition

- To impose a monotonicity condition on the time series {*w*_*i*_(*t*)}, a new variable η_*i*_(*t*) is defined as follows:

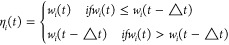


4. Autocorrelation function calculation

- Using η_*i*_(*t*), the autocorrelation function *C*_*i*_^*M*^(τ) is computed, where *M* is the water model used in the simulations:

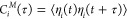


5. Time-averaged autocorrelation function

- The time-averaged autocorrelation function for each residue, *i*, is computed:

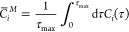


6. Standardization and scaling

- The PARCH value for water model *M* is standardized by normalizing  against the time-averaged autocorrelation function of a reference zwitterionic single amino residue, .

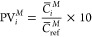

- Since the quantity, , ranges between 0 and 1, multiplying by 10 expands the PARCH values onto a broader 0–10 scale, as shown.

## 3 Methods

### 3.1 Protein Structure and Equilibration

We selected 14 proteins (Table 2) for this study, previously reported in our earlier work.^9^ The proteins were modeled using the CHARMM36m force field,^12^ while four water models—TIP3P, TIP4P, TIP4-Ew, and TIP5P—were employed to represent the solvent environment. Simulations were conducted using GROMACS 2023.2.^25^ Each protein was solvated in a 0.15 M NaCl solution, and the solvated systems underwent energy minimization using the steepest descent algorithm with a 10^3^ kJ mol^–1^ force tolerance. Following minimization, the systems were equilibrated under isothermal–isochoric (*NVT*) and isothermal–isobaric (*NPT*) conditions for 2 ns each at 300 K, with a 2 fs time step. During the *NVT* equilibration, position restraints were applied to the protein’s heavy (nonhydrogen) atoms with a force constant of 400 kJ mol^–1^ nm^–2^. The restraint was reduced to 40 kJ mol^–1^ nm^–2^ during *NPT* equilibration to facilitate gradual structural relaxation. Electrostatic and van der Waals interactions were calculated with a 1.2 nm cutoff. The Particle–Mesh Ewald (PME) method^26,27^ was employed to handle long-range electrostatic interactions with a 1.2 nm cutoff. Temperature regulation was achieved using the v-rescale thermostat^28^ with a coupling constant of 1.0 ps. During the *NPT* equilibration, isotropic pressure was maintained at 1 bar using the Berendsen barostat,^29^ with a pressure coupling constant of 5.0 ps and a compressibility of 4.5 × 10^–5^ bar^–1^. Hydrogen-related bonds were constrained using the linear constraint solver (LINCS) algorithm.^30^ Finally, a 100 ns production run was carried out under *NPT* conditions using the Parrinello–Rahman barostat^31^ without position restraints to enable equilibration of the protein backbone and side chains.

**Table 2:** List of Proteins Studied

protein	PDB ID	monomer (M)/dimer (D)	function
bacteriophage T4 lysozyme (LYM)	253L^32^	M	soluble, enzyme
thymidylate synthase (TS)	2TSC^33^	M	soluble, enzyme
malate dehydrogenase (MDH)	3HHP^34^	M	soluble, enzyme
barnase (BNS)^a^	1BRS^35^	M	soluble, enzyme
mouse double minute 2 (MDM2)	1YCR^36^	M	soluble, enzyme
mannose-binding protein (MBP)^b^	1MSB^37^	M	soluble, plasma, immune
		D	
hydrophobin II (HP2)	2B97^38^	M	soluble, structural
hepatitis B viral capsid (HBV)	1QGT^39^	M	soluble, viral capsid, structural
		D	
melittin (MLT)	2MLT	D	membrane-active
claudin-5 (CLD5)^c^	(40)	M	membrane
aquaporin (AQP1)	1J4N^41^	M	membrane
ghrelin *O*-acyltransferase (hGOAT)^c^	(42)	M	membrane, enzyme

a In the absence of barstar.

b In the absence of p53.

c Published computational model.

### 3.2 Simulation Setup for PARCH Calculations

**Figure 1:**
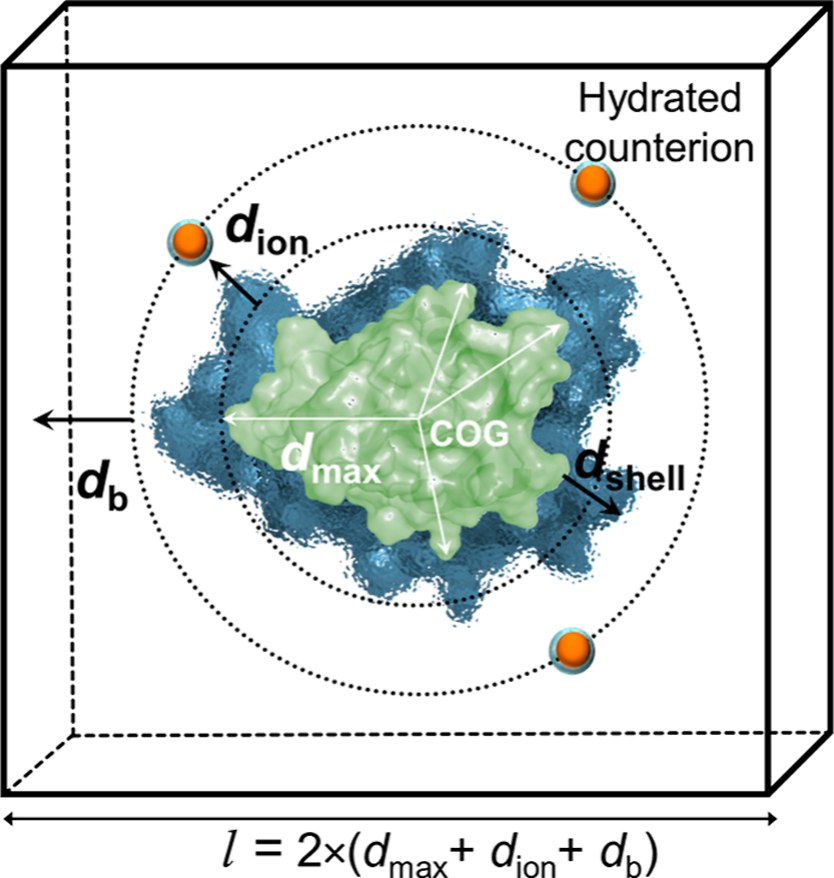
Schematic of the PARCH scale setup. The protein (green) of maximum radius (*d*_max_) is solvated in an explicit water shell (*d*_shell_) with hydrated counterions (orange spheres) at a fixed distance (*d*_ion_) from protein surface. The cell boundary is at a radius (*d*_b_) from the counterions, deciding the final box dimension *l*.

We followed the established PARCH calculation protocol for this work^9^ (Figure 1). Each protein was studied with four water models. PARCH calculations were also performed for the 20 zwitterionic amino acids for each water model. The molecular structures of the water models and the associated force field parameters were obtained from the libraries available in GROMACS 2023.2^25^. As in previous studies, we computed the radial distribution functions (RDFs) to define the thickness of the water shell for each water model. The RDFs of the protein backbone’s C_α_ atom and water’s oxygen atom consistently yielded a shell thickness *d*_shell_ = 0.415 (Figure S2), corresponding to the second hydration shell. Other key parameters, including the counterion–protein surface distance (*d*_ion_ = 3 nm) and ion–box boundary distance (*d*_b_ = 3 nm), remained unchanged from our original PARCH protocol. Hydrated counterions were placed based on the distance *d*_ion_ from the protein surface to balance the net charge of the system. The counterions were also position-restrained during the annealing to ensure stability and reproducibility of the PARCH calculations.

The systems were energy-minimized using the steepest descent algorithm with 10^3^ kJ mol^–1^ force tolerance and simulated in the *NVT* ensemble condition with increasing temperatures using the annealing protocol available in GROMACS. The temperature of the system was increased from 300 to 800 K at a rate of 1 K/10 ps during the annealing process, consistent with our original protocol. Each system was performed in quintuplicate to ensure that PARCH values were sampled well. We used a consistent cutoff for water molecules contacting a residue (*d*_water_ = 0.315 nm) for the four water models to quantify and scale the PARCH values.

### 3.3 Impact of the Position Restraint Force Constant during the Annealing Process

We tested another three values of the position restraint force constants (5 × 10^3^, 1 × 10^4^, and 2 × 10^4^ kJ mol^–1^ nm^–2^) and compared the PARCH values to the original values obtained using the 10^3^ kJ mol^–1^ nm^–2^ force constant. The results and interpretation of this exercise are discussed in the next section.

### 3.4 Data Visualization

The average PARCH values for the quintuple runs were written in the out file in the protein databank (PDB) format as b-factors. The variances of PARCH values resulting from water models were calculated relative to TIP3P on each residue site, and the variance distributions were analyzed according to the types of residues.

## 4 Results and Discussion

### 4.1 Lysine Is a Reference Residue for PARCH Scale Calculations across Multiple Water Models

A critical step in computing PARCH values is identifying a reference residue, defined as the amino acid with the highest time-averaged autocorrelation function, , among the 20 zwitterionic amino acids. The autocorrelation curves for the single zwitterionic amino acids obtained using the TIP3P water model are shown in Figure S3.

The water force field parameters—such as partial charges, virtual sites, and bonded and nonbonded interaction parameters—can influence the choice of the reference residue. Consequently, each water model could, in principle, have a different reference amino acid. For all four water models (TIP3P, TIP4P, TIP4P-Ew, and TIP5P), charged residues generally exhibit higher  values.

The relative ordering of amino acids based on increasing  values varies across water models. For instance, in the TIP5P water model, the PARCH value of phenylalanine (PHE) is lower than that of alanine (ALA), suggesting that PHE is more hydrophobic than ALA. In contrast, this ordering is reversed in the other three water models, highlighting how the choice of water model influences the water retention behavior of zwitterionic amino acids.

Despite these differences, lysine consistently shows the highest  value across all four water models, making it a reliable reference residue. Thus,  was selected as the benchmark for PARCH calculations in each model. Table 3 presents the PARCH values for the 20 single amino acids across all water models, with lysine consistently assigned a PARCH value of 10 in every case.

**Table 3:**
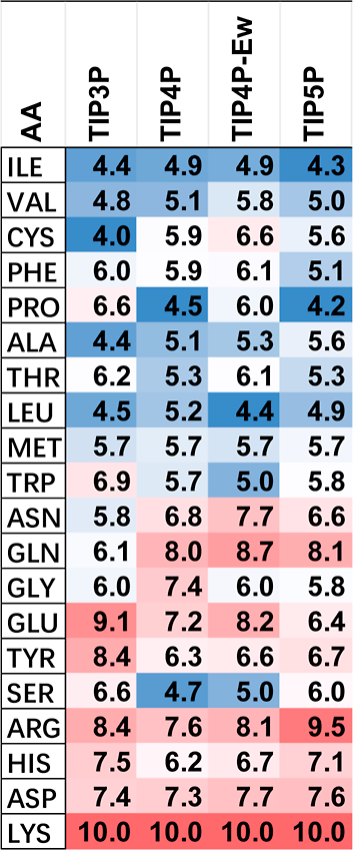
PARCH Values of the 20 Zwitterionic Amino Acids Computed Using the Four Water Models^a^

a For each water model, the PARCH values are color-coded from minimum (blue) to maximum (red).

### 4.2 TIP3P, TIP4P, TIP4P-Ew, and TIP5P Water Models Can Be Used to Compute the Hydropathy of Proteins

To assess the role of water models in hydropathy calculations, we categorized 14 proteins into integral membrane proteins, soluble proteins, and self-associating proteins, which exhibit distinct hydropathy profiles.

### 4.3 Integral Membrane Proteins

**Figure 2:**
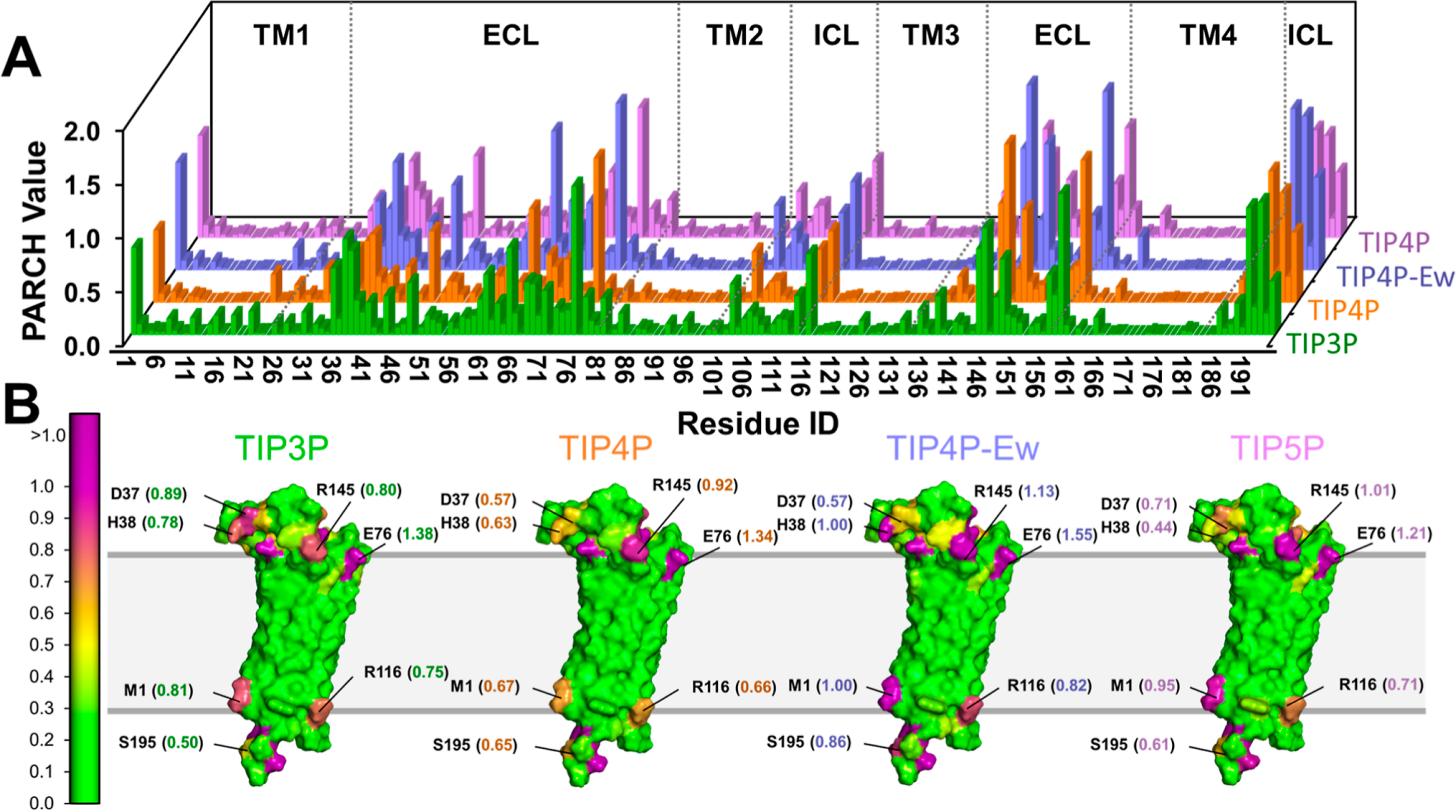
Hydropathy of claudin-5 (CLD5) for different water models. (A) PARCH value profiles of CLD5 for water models: TIP3P (green), TIP4P (orange), TIP4P-Ew (blue), and TIP5P (violet). (B) Protein surface representations illustrating hydropathy distributions across water models. Residues are colored based on PARCH values with the scale shown on the left. Residue identities and PARCH values (in parentheses) are labeled.

The transmembrane domains of membrane proteins are hydrophobic, enabling them to traverse the lipid bilayer’s hydrophobic core. Claudin-5, a key protein in tight junction formation, contains four transmembrane domains (TM1–TM4) spanning residues G2–G26, A82–A102, V117–C136, and G161–G189, respectively. Analysis of PARCH values across all four water models revealed consistently low values (<0.3) for the TM1–TM4 domains (Figure 2A), reflecting their hydrophobic nature.

In contrast, the extracellular loops (ECLs) and intracellular loops (ICLs), which are water-accessible and enriched with charged residues, exhibited higher PARCH values. These regions include residues such as D37, K48, K65, D68, E76, R116, R145, E146, R191, D149, K157, and D193 and the charged termini. Across all water models, claudin-5 consistently displayed a distinct hydrophobic zone corresponding to its transmembrane domains embedded within the lipid membrane (Figure S4). The top and bottom regions, rich in hydrophilic residues, represent the ECLs and ICLs that interact with the aqueous environment. Residues at these lipid–aqueous interfaces exhibit the largest variations in PARCH values. For example, H38 has a PARCH value of 0.44 in TIP5P but increases to 1.00 in TIP4P-Ew. Similarly, R145 displays values ranging from 0.80 to 1.13 across the four water models (Figure 2B). Despite these variations in PARCH values for specific residues, all four water models can be used to predict the hydropathy of claudin-5.

We extended this analysis to other membrane proteins, including the melittin dimer, MLT (Figure S5), aquaporin-1, AQP1 (Figure S6), and human ghrelin *O*-acyltransferase, hGOAT (Figure S7). In all cases, the PARCH scale effectively distinguished the separation of hydrophobic and hydrophilic zones in the four water models, demonstrating its utility for characterizing protein–lipid and protein–water interactions.

### 4.4 Soluble Proteins

**Figure 3:**
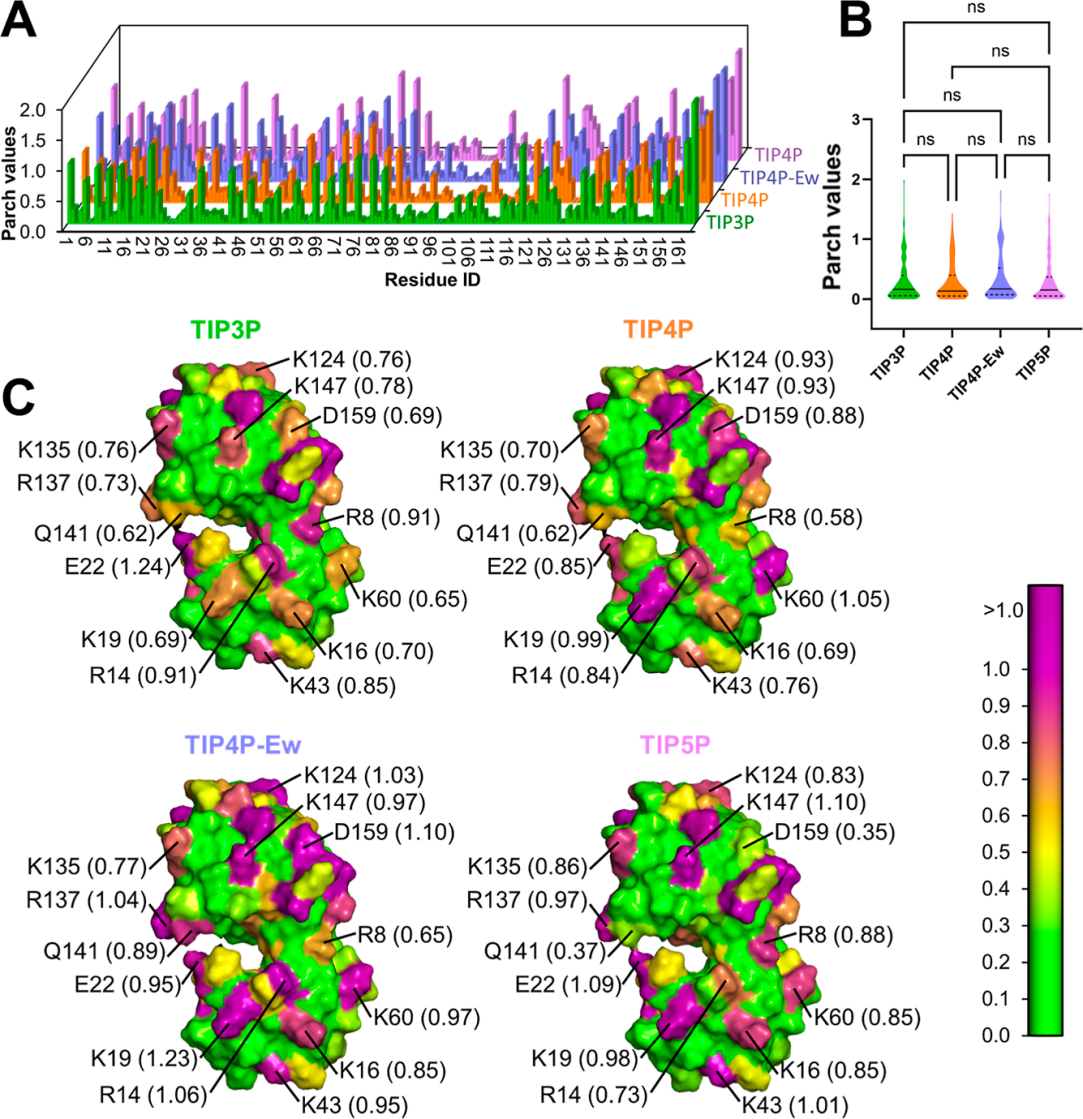
Hydropathy of lysozyme (LYM) for different water models. (A) PARCH value profiles of LYM for water models: TIP3P (green), TIP4P (orange), TIP4P-Ew (blue), and TIP5P (violet). (B) The PARCH values distributions of residues on LYM show no statistically significant difference between water models. (C) Protein surface representations illustrating hydropathy distributions across water models. Residues are colored based on PARCH values with the scale on the right. Residue identities and PARCH values (in parentheses) are labeled.

We evaluated the chloride values of bacteriophage lysozyme T4 (LYM) as a representative soluble protein (Figure 3). Of the 164 residues in LYM, 43 are charged and distributed across the protein’s surface, enabling solvent accessibility and facilitating binding to the bacterial peptidoglycan layer. As a result, the segregation of hydrophobic and hydrophilic zones is less pronounced compared to membrane proteins, a characteristic common among soluble proteins. The trend of residue PARCH values along the amino acid sequence is consistent across the four water models, with the PARCH scale effectively capturing the relatively high hydropathy values for abundantly charged residues like lysine, arginine, and glutamic acid. High PARCH values are observed across most of the protein surface, except for a region around residues L91–M106 (Figures 3A and S8), which are buried in the protein’s interior and have limited water accessibility.

Statistical analysis revealed no significant differences in the overall PARCH values for LYM across the four water models (Figure 3B). However, residue-specific comparisons (Figure 3C) showed patch value variations of ±0.3 for charged surface residues depending on the water model. These variations are expected for soluble proteins due to their strong interactions with water. Differences in water models—such as the inclusion of a virtual site in TIP4P, the electrostatic Ewald summation in TIP4P-Ew, or lone pair representations in TIP5P—affect local interactions with proteins and, consequently, the PARCH values. These local variations highlight the sensitivity of the PARCH approach to water model changes while demonstrating its robustness in accurately capturing the overall hydropathy trends of the protein.

Beside LYM, we also examined parameter values of enzymes TS (Figure S9), MDH (Figure S10), and BNS (Figure S11). The topographical details together with hydropathy will result in distinct regions like hydrophobic patches, which play an important role in guiding protein assembly. The use of different water models maintains the accuracy of PARCH in capturing these properties effectively.

### 4.5 Self-Associating and Binding Proteins

Protein dimerization and oligomerization are essential processes that regulate protein function, structure, and activity while optimizing genome size through modular complex formation. To analyze the influence of water models on such proteins, we included a selection of proteins that assemble via hydrophobic patches or exhibit sufficient hydrophobicity to interact with other biomolecules in the cell. Unlike soluble proteins, these proteins are characterized by hydrophobic regions or patches on their surfaces and lack the enrichment of charged residues.

**Figure 4:**
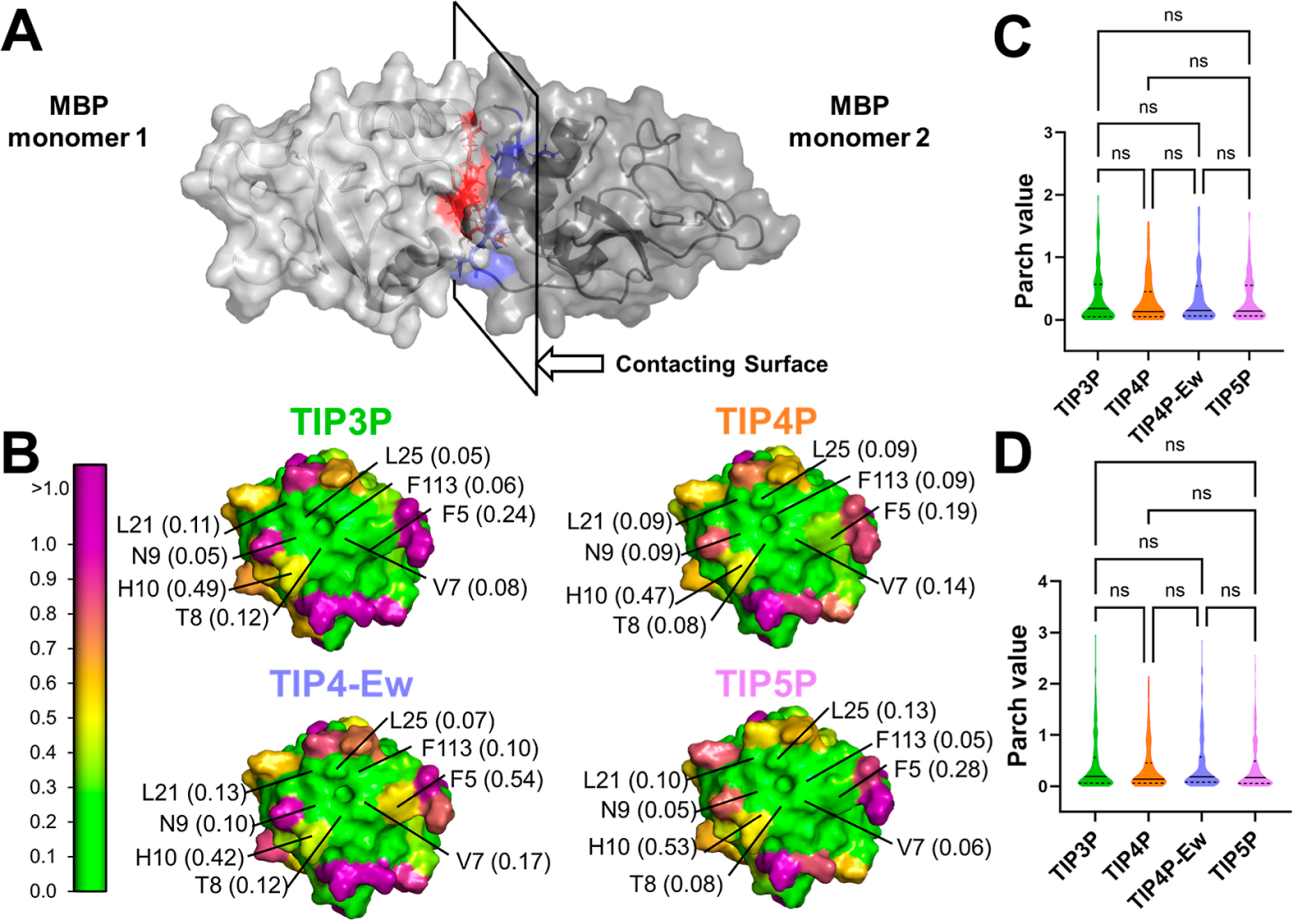
Hydropathy of mannose-binding protein (MBP) across different water models. (A) Surface representation of the MBP dimer, formed by head-to-head interaction of contacting regions in red and blue. (B) Zoomed-in view of the contacting regions, shown with surface representations across different water models. Residues are color-coded based on the PARCH scale provided on the left. Key residues and their PARCH values (in parentheses) are labeled for clarity. The PARCH data consistently captures the hydrophobic patch critical for dimerization across all water models. (C) Statistical analysis of PARCH values shows no significant differences in the residue distributions in (C) monomeric (MBPm) and (D) dimeric (MBPd) across the water models.

In this category, we first analyzed the PARCH values of mannose-binding protein (MBP) in its monomeric (MBPm) and dimeric (MBPd) forms (Figure 4). MBP forms a dimer by interacting through a flat hydrophobic surface (Figure 4A). Across all four water models, the contacting residues of MBPm—including V7, T8, N9, L21, L25, and F113—consistently exhibited PARCH values below 0.2, indicating a strong hydrophobic character critical for MBP dimerization (Figure 4B). In contrast, peripheral residues on the flat surface, such as H10, showed higher PARCH values, contributing to a hydrophilic, water-exposed surface in the dimer.

A detailed comparison of gap values between the monomeric and dimeric states is provided in the Supporting Information (Figures S12 and S13). Remarkably, PARCH data across all four water models produced a consistent hydropathy landscape for the MBPm structure. Statistical analysis revealed no significant differences in PARCH values for MBPm and MBPd across the four water models (Figures 4C, D), highlighting the robustness of PARCH in representing the hydropathy profiles of MBP in both states.

Additionally, similar analyses using PARCH to identify hydrophobic patches facilitating the dimerization of the hepatitis B viral capsid (HBVm and HBVd, Figures S14 and S15) and binding interactions with other proteins, such as hydrophobin II (HP2, Figure S16) and Mouse double minute 2 (MDM2, Figure S17), demonstrated consistent results across all water models. These findings underscore the capability of the PARCH scale to predict hydrophobic patches essential for protein assembly, reaffirming its utility in studying protein interactions and complex formation.

**Figure 5:**
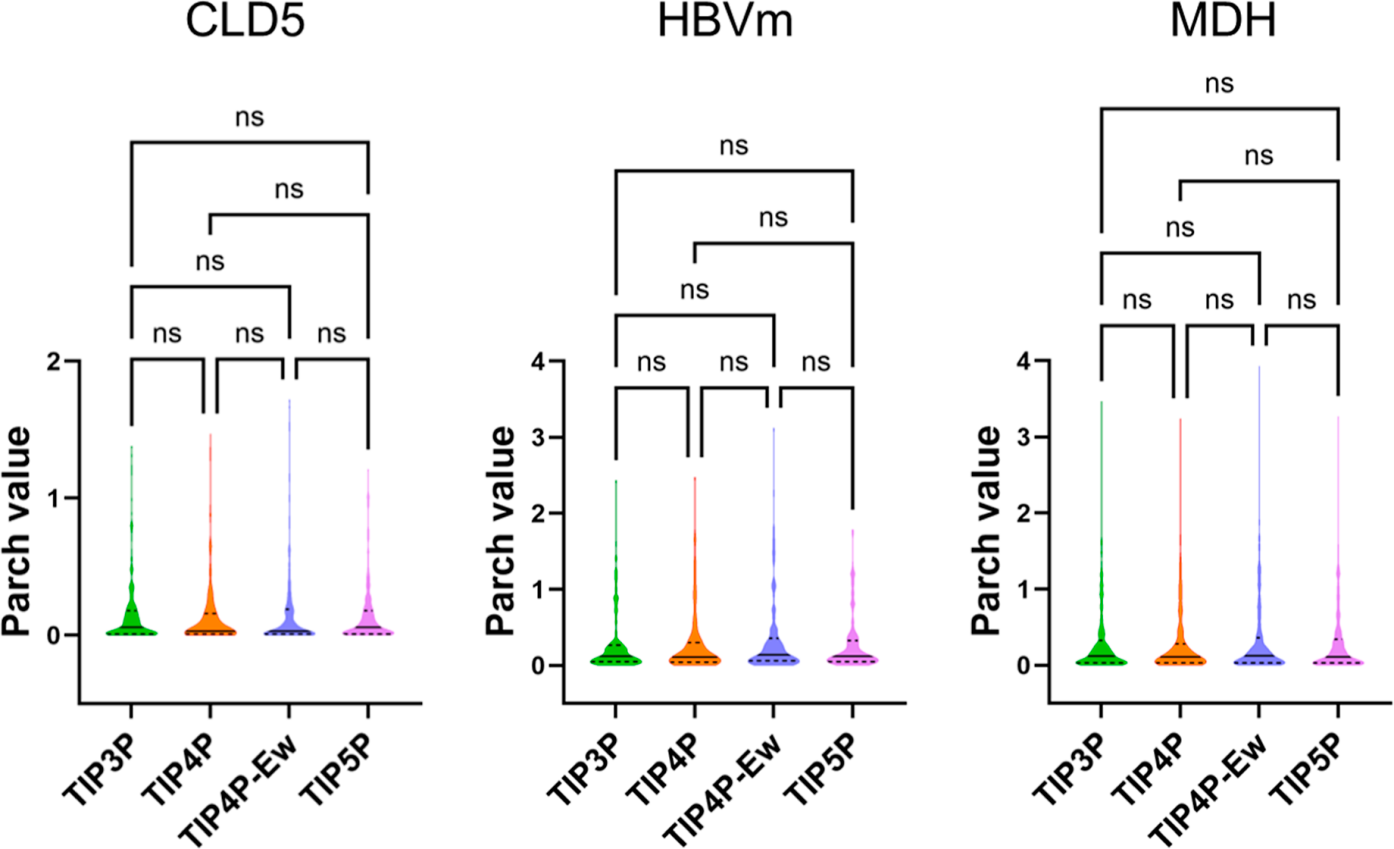
Violin plots comparing PARCH values of three proteins across four water models: TIP3P, TIP4P, TIP4P-Ew, and TIP5P. The colors indicate different water models. The label “ns” indicates not statistically significant for *p* > 0.05, while *, **, and *** indicate statistically significant differences of *p* ≤ 0.05, *p* ≤ 0.01, and *p* ≤ 0.001, respectively.

Finally, we statistically compared the PARCH value distributions for each of the 14 proteins across all four water models (Figures 5 and S18). The analysis revealed no significant differences (*p* > 0.05) among the water models. This consistency indicates that all four water models produce comparable hydropathy landscapes, regardless of the protein’s structure or function.

### 4.6 Impact of Water Model Choice on PARCH Values of Charged Amino Acids

Based on our observation of the charged surface residues in soluble proteins, for example, LYM (Figure 3), we pooled the PARCH data of all 2753 residues in the 14 proteins and evaluated the PARCH value distribution of the 20 amino acids across the four water models. The rationale for this grouping was to determine if some amino acids were more sensitive to the choice of the water model.

### 4.7 Residue-Specific Variations across Water Models

**Figure 6:**
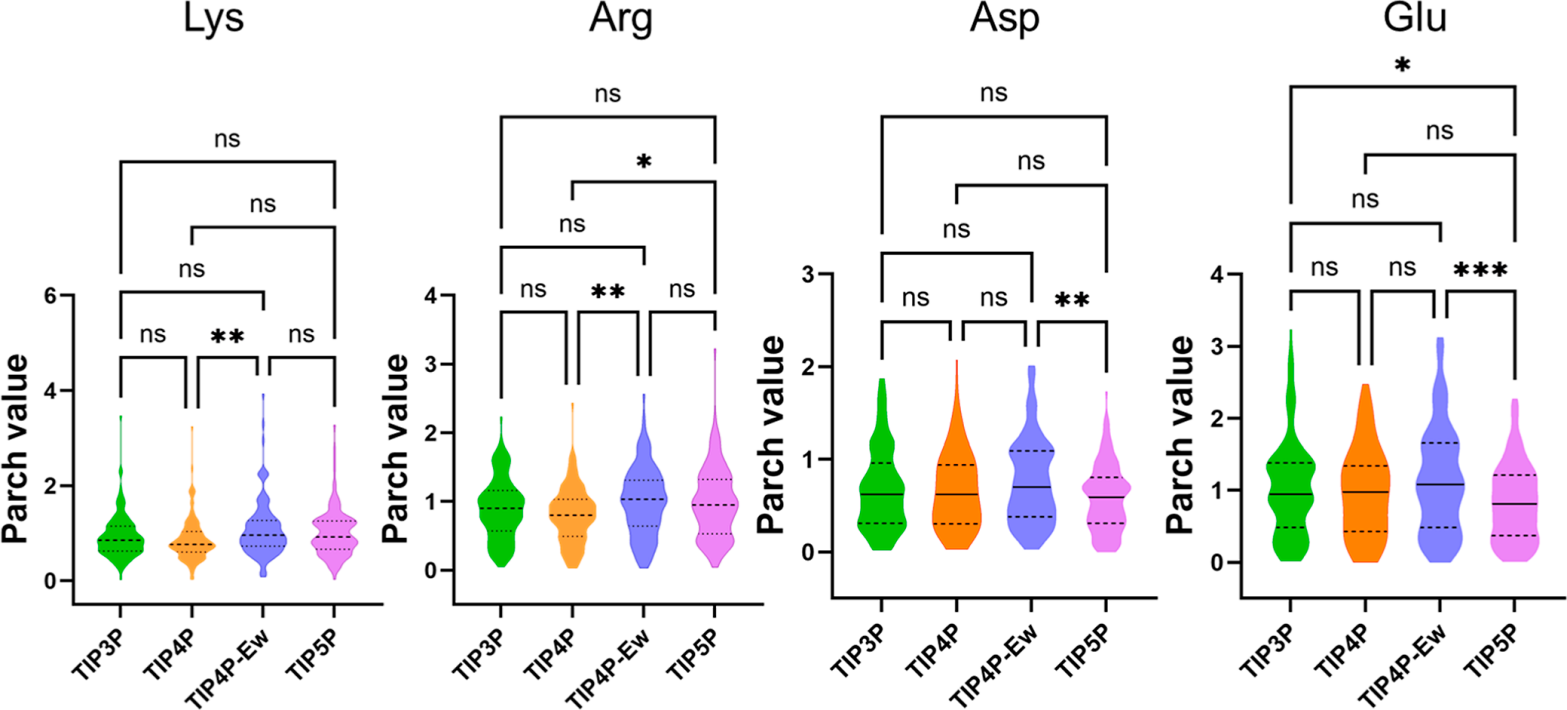
Violin plots comparing PARCH values of charged amino acids across four water models: TIP3P, TIP4P, TIP4P-Ew, and TIP5P. The colors indicate different water models. The label “ns” indicates not statistically significant for *p* > 0.05, while *, **, and *** indicate statistically significant differences of *p* ≤ 0.05, *p* ≤ 0.01, and *p* ≤ 0.001, respectively.

The PARCH value distributions for each residue type were categorized by residues and compared across other water models (Figures 6 and S19). Notably, charged residues—lysine, arginine, aspartic acid, and glutamic acid—showed some differences across the water models, suggesting that the charged residues are more influenced by water model parameters because the distribution of charges on water molecules impacts the nonbonded interactions.

For lysine, arginine, aspartic acid, and glutamic acid, the median variance in PARCH values relative to TIP3P showed minor offsets (no greater than ±0.17, with most values below ±0.1). However, these residues exhibited broad distributions of variance, indicating variability in their percentages across water models. Residues like histidine, tryptophan, tyrosine, glutamine, and asparagine also showed less concentrated variance distributions, likely due to their long, hydrogen-bonding-capable side chains, which make them more susceptible to water model variations.

In contrast, residues with short or nonpolar side chains (Figure S19) displayed medians of variance at zero and more concentrated distributions, suggesting minimal differences in PARCH values across water models. These residues are less sensitive to water model differences due to their simpler interactions with the solvent.

### 4.8 PARCH Approach Robustness

The PARCH value is computed based on the number of water molecules remaining in contact with a residue during annealing, which depends on both the residue’s chemical properties and topology. Although absolute PARCH values for individual residues may vary with the water model, relative PARCH values remain consistent, preserving the overall hydropathy trends of the proteins.

Statistical analysis revealed no significant variances for most residues. Exceptions included lysine (between TIP4P and TIP4P-Ew), arginine (between TIP4P and TIP4P-Ew and TIP4P and TIP5P), aspartate (between TIP4P-Ew and TIP5P), and glutamic acid (between TIP3P and TIP5P and TIP4P-Ew and TIP5P), highlighting minor sensitivities to water model differences. The most significant difference observed was for glutamic acid TIP4P-Ew and TIP5P. Despite these exceptions, the PARCH scale consistently captured the hydropathy of residues and proteins, regardless of water model choice.

### 4.9 Hydropathy Trends across Water Models

By investigating PARCH values across different water models, we demonstrated that the PARCH approach effectively identified relative hydropathy distributions in proteins. While individual residue PARCH values may vary depending on the water model, the overall trends and segregation of hydrophobic and hydrophilic regions remain consistent. All water models accurately captured the hydropathy landscape of proteins, emphasizing that hydrophobic and hydrophilic regions—determined by both chemical properties and topology—can be reliably identified using the PARCH approach across diverse water models.

### 4.10 Insights on Time Step and Position Restraints of the PARCH Scale

**Figure 7:**
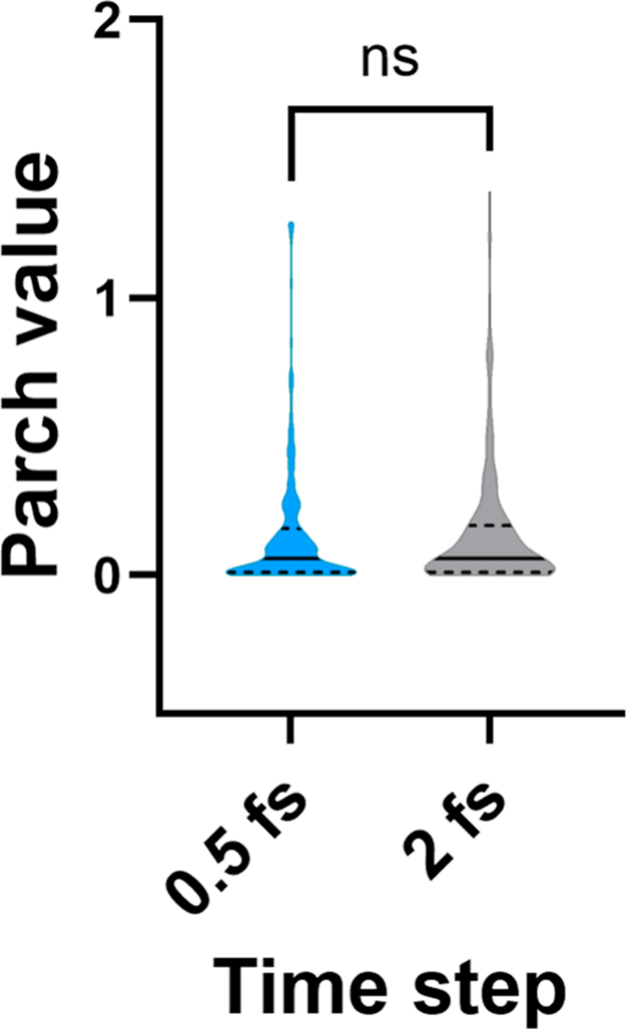
PARCH value (PV) distribution using time steps of 0.5 and 2 fs.

A recently published study reported that using a 0.5 fs time step, which is smaller than the typical 2 fs used in molecular dynamics simulations, improves the equilibration of water molecules.^43^ To investigate the effect of this smaller time step on PARCH scale calculations, we conducted simulations for claudin-5 using the TIP3P water model. The simulations employed an annealing rate of 1 K/10 ps, ranging from 300 to 800 K, with 0.5 and 2 fs time steps. Our results showed that changing the time step had no significant impact on the distribution of PARCH values (Figure 7). However, the use of a smaller time step (0.5 fs) increased the computational cost of the PARCH calculation by approximately 4-fold.

We further examined the effect of position restraints on the PARCH scale by calculating the root-mean-square deviation (RMSD) for hGOAT and MLT proteins (Figure S20A,B), the largest and smallest proteins among the fourteen studied. At position restraints of 1 × 10^3^ kJ mol^–1^ nm^–2^, the RMSD remained below 0.07 nm during standard equilibration simulations, indicating stability. However, during the PARCH annealing process, where the temperature increases, RMSD values became larger and more fluctuated at higher temperatures, suggesting decreased protein stability. These fluctuations highlight the sensitivity of protein stability to temperature fluctuations during annealing.

Since the PARCH scale considers structural conformations, changes in amino acid side-chain configurations during the annealing process can influence water evaporation and the PARCH values. To mitigate this, we tested the effect of increasing the position restraint force constant to 5 × 10^3^, 10 × 10^3^, and 20 × 10^3^ kJ mol^–1^ nm^–2^. Higher restraint values resulted in lower RMSD values, indicating improved protein stability (Figure S20C). However, the improvement in stability between 10^4^ and 2 × 10^4^ kJ mol^–1^ nm^–2^ was relatively minor. Based on these findings, a force constant of 1 × 10^4^ kJ mol^–1^ nm^–2^ was selected to optimize stability without introducing unnecessary computational overhead.

## 5 Conclusions

In this study, we expanded the original PARCH scale by incorporating three additional water models—TIP4P, TIP4P-Ew, and TIP5P—alongside TIP3P to investigate protein hydropathy. Through the PARCH analysis of 14 proteins with diverse structures and functions, we demonstrate that the overall distribution of PARCH hydropathy is highly consistent across all four water models. The chemical properties of amino acid residues are captured reliably, with polar or charged residues such as lysine, arginine, aspartic acid, and glutamic acid typically exhibiting higher PARCH values. Hydrophobic regions, such as transmembrane domains, are clearly distinguishable from hydrophilic regions, such as extracellular and intracellular loops. Furthermore, topology information is accurately captured across all water models as residues buried in the protein’s core or less solvent-accessible regions exhibit lower PARCH values compared to those in water-accessible regions. However, absolute PARCH values for individual residues may vary depending on the water model, with charged residues showing greater variability. Additionally, residues with side chains capable of hydrogen bonding, such as histidine, tryptophan, tyrosine, glutamine, and asparagine, display sensitivity to different water models. In contrast, residues with nonpolar or small side chains show minimal variance in PARCH values across the models. This study confirms the robustness of the PARCH scale in calculating protein hydropathy using any of the four water models, facilitating its application across diverse protein–water force field combinations and even extending its use to molecular systems beyond proteins.

## Data Availability

The data set for all structures and computed PARCH values for the protein is available at https://github.com/NangiaLab/PARCH-water-models. There is no restriction on the use of the data.

## Supporting Information Available

Radial distribution functions of water around proteins, statistical comparison of PARCH values of amino acids, statistical comparison of PARCH values of 14 proteins, and PARCH value profiles of 14 proteins across TIP4P, TIP4-Ew, and TIP5P water models (PDF)

## References

[ref1] Cui D.; Ou S. C.; Patel S. Protein-Spanning Water Networks and Implications for Prediction of Protein-Protein Interactions Mediated through Hydrophobic Effects. Proteins 2014, 82, 3312–3326. 10.1002/prot.24683. 25204743

[ref2] Giovambattista N.; Lopez C. F.; Rossky P. J.; Debenedetti P. G. Hydrophobicity of Protein Surfaces: Separating Geometry from Chemistry. Proc. Natl. Acad. Sci. USA 2008, 105, 2274–2279. 10.1073/pnas.0708088105. 18268339 PMC2268126

[ref3] Hummer G.; Garde S.; García A. E.; Pratt L. R. New Perspectives on Hydrophobic Effects. Chem. Phys. 2000, 258, 349–370. 10.1016/S0301-0104(00)00115-4.

[ref4] Wang X.; Zhang Y. Q.; Yu B.; Salhi A.; Chen R. X.; Wang L.; Liu Z. F. Prediction of Protein-Protein Interaction Sites through Extreme Gradient Boosting with Kernel Principal Component Analysis. Comput. Biol. Med. 2021, 134, 104516 10.1016/j.compbiomed.2021.104516. 34119922

[ref5] Xia X. H.; Li W. H. What Amino Acid Properties Affect Protein Evolution?. J. Mol. Evol. 1998, 47, 557–564. 10.1007/PL00006412. 9797406

[ref6] Irudayanathan F. J.; Trasatti J. P.; Karande P.; Nangia S. Molecular Architecture of the Blood Brain Barrier Tight Junction Proteins-a Synergistic Computational and Approach. J. Phys. Chem. B 2016, 120, 77–88. 10.1021/acs.jpcb.5b09977. 26654362

[ref7] Rego N. B.; Xi E. T.; Patel A. J. Identifying Hydrophobic Protein Patches to Inform Protein Interaction Interfaces. Proc. Natl. Acad. Sci. USA 2021, 118, e2018234118 10.1073/pnas.2018234118. 33526682 PMC8018078

[ref8] Shinoda S.; Itakura A.; Sasano H.; Miyake R.; Kawabata H.; Asano Y. Rational Design of the Soluble Variant of L-Pipecolic Acid Hydroxylase Using the Α-Helix Rule and the Hydropathy Contradiction Rule. ACS Omega 2022, 7, 29508–29516. 10.1021/acsomega.2c04247. 36033675 PMC9404520

[ref9] Ji J. J.; Carpentier B.; Chakraborty A.; Nangia S. An Affordable Topography-Based Protocol for Assigning a Residue’s Character on a Hydropathy (Parch) Scale. J. Chem. Theory Comput. 2024, 20, 1656–1672. 10.1021/acs.jctc.3c00106. 37018141 PMC10902853

[ref10] Ji J. J.; Shukla A. D.; Mandal R.; Khondkar W. I.; Mehl C. R.; Chakraborty A.; Nangia S. Nanoscale Topography Dictates Residue Hydropathy in Proteins. Langmuir 2024, 40, 22049–22057. 10.1021/acs.langmuir.4c02142. 39392451 PMC11500397

[ref11] Jorgensen W. L.; Chandrasekhar J.; Madura J. D.; Impey R. W.; Klein M. L. Comparison of Simple Potential Functions for Simulating Liquid Water. J. Chem. Phys. 1983, 79, 926–935. 10.1063/1.445869.

[ref12] Huang J.; Rauscher S.; Nawrocki G.; Ran T.; Feig M.; de Groot B. L.; Grubmüller H.; MacKerell A. D. Charmm36m: An Improved Force Field for Folded and Intrinsically Disordered Proteins. Nat. Methods 2017, 14, 71–73. 10.1038/nmeth.4067. 27819658 PMC5199616

[ref13] Horn H. W.; Swope W. C.; Pitera J. W.; Madura J. D.; Dick T. J.; Hura G. L.; Head-Gordon T. Development of an Improved Four-Site Water Model for Biomolecular Simulations: Tip4p-Ew. J. Chem. Phys. 2004, 120, 9665–9678. 10.1063/1.1683075. 15267980

[ref14] Mahoney M. W.; Jorgensen W. L. A Five-Site Model for Liquid Water and the Reproduction of the Density Anomaly by Rigid, Nonpolarizable Potential Functions. J. Chem. Phys. 2000, 112, 8910–8922. 10.1063/1.481505.

[ref15] Vega C.; Abascal J. L. F. Simulating Water with Rigid Non-Polarizable Models: A General Perspective. Phys. Chem. Chem. Phys. 2011, 13, 19663–19688. 10.1039/c1cp22168j. 21927736

[ref16] Chan R.; Falato M.; Liang H. Y.; Chen L. Y. *in Silico* Simulations of Erythrocyte Aquaporins with QuantitativE *in Vitro* Validation. RSC Adv. 2020, 10, 21283–21291. 10.1039/D0RA03456H. 32612811 PMC7328926

[ref17] Nutt D. R.; Smith J. C. Molecular Dynamics Simulations of Proteins: Can the Explicit Water Model Be Varied?. J. Chem. Theory Comput. 2007, 3, 1550–1560. 10.1021/ct700053u. 26633225

[ref18] Emperador A.; Crehuet R.; Guàrdia E. Effect of the Water Model in Simulations of Protein–Protein Recognition and Association. Polymers 2021, 13, 176 10.3390/polym13020176. 33419008 PMC7825341

[ref19] Florová P.; Sklenovský P.; Banáš P.; Otyepka M. Explicit Water Models Affect the Specific Solvation and Dynamics of Unfolded Peptides While the Conformational Behavior and Flexibility of Folded Peptides Remain Intact. J. Chem. Theory Comput. 2010, 6, 3569–3579. 10.1021/ct1003687. 26617103

[ref20] Glass D. C.; Krishnan M.; Nutt D. R.; Smith J. C. Temperature Dependence of Protein Dynamics Simulated with Three Different Water Models. J. Chem. Theory Comput. 2010, 6, 1390–1400. 10.1021/ct9006508.

[ref21] Thirunavukarasu A. S.; Szleper K.; Tanriver G.; Marchlewski I.; Mitusinska K.; Gora A.; Brezovsky J. Water Migration through Enzyme Tunnels Is Sensitive to the Choice of Explicit Water Model. J. Chem. Inf. Model. 2024, 65, 326–337. 10.1021/acs.jcim.4c01177. 39680044 PMC11733929

[ref22] Izadi S.; Onufriev A. V. Accuracy Limit of Rigid 3-Point Water Models. J. Chem. Phys. 2016, 145, 074501 10.1063/1.4960175. 27544113 PMC4991989

[ref23] Netz P. A. Molecular Dynamics Simulations of Structural and Dynamical Aspects of DNA Hydration Water. J. Phys.: Condens. Matter 2022, 34, 164002 10.1088/1361-648X/ac5198. 35114661

[ref24] Shi R.; Tanaka H. Microscopic Structural Descriptor of Liquid Water. J. Chem. Phys. 2018, 148, 124503 10.1063/1.5024565. 29604883

[ref25] Abraham M. J.; Murtola T.; Schulz R.; Páll S.; Smith J. C.; Hess B.; Lindahl E. Gromacs: High Performance Molecular Simulations through Multi-Level Parallelism from Laptops to Supercomputers. SoftwareX 2015, 1–2, 19–25. 10.1016/j.softx.2015.06.001.

[ref26] Darden T.; York D.; Pedersen L. Particle Mesh Ewald - an N.Log(N) Method for Ewald Sums in Large Systems. J. Chem. Phys. 1993, 98, 10089–10092. 10.1063/1.464397.

[ref27] Essmann U.; Perera L.; Berkowitz M. L.; Darden T.; Lee H.; Pedersen L. G. A Smooth Particle Mesh Ewald Method. J. Chem. Phys. 1995, 103, 8577–8593. 10.1063/1.470117.

[ref28] Bussi G.; Donadio D.; Parrinello M. Canonical Sampling through Velocity Rescaling. J. Chem. Phys. 2007, 126, 014101 10.1063/1.2408420. 17212484

[ref29] Berendsen H. J. C.; Postma J. P. M.; Vangunsteren W. F.; Dinola A.; Haak J. R. Molecular-Dynamics with Coupling to an External Bath. J. Chem. Phys. 1984, 81, 3684–3690. 10.1063/1.448118.

[ref30] Hess B.; Bekker H.; Berendsen H. J. C.; Fraaije J. G. E. M. Lincs: A Linear Constraint Solver for Molecular Simulations. J. Comput. Chem. 1997, 18, 1463–1472. 10.1002/(SICI)1096-987X(199709)18:12<1463::AID-JCC4>3.0.CO;2-H.

[ref31] Parrinello M.; Rahman A. Polymorphic Transitions in Single-Crystals - a New Molecular-Dynamics Method. J. Appl. Phys. 1981, 52, 7182–7190. 10.1063/1.328693.

[ref32] Shoichet B. K.; Baase W. A.; Kuroki R.; Matthews B. W. A Relationship between Protein Stability and Protein Function. Proc. Natl. Acad. Sci. USA 1995, 92, 452–456. 10.1073/pnas.92.2.452. 7831309 PMC42758

[ref33] Montfort W. R.; Perry K. M.; Fauman E. B.; Finermoore J. S.; Maley G. F.; Hardy L.; Maley F.; Stroud R. M. Structure, Multiple Site Binding, and Segmental Accommodation in Thymidylate Synthase on Binding Dump and an Antifolate. Biochemistry 1990, 29, 6964–6977. 10.1021/bi00482a004. 2223754

[ref34] Zaitseva J.; Meneely K. M.; Lamb A. L. Structure of Escherichia Coli Malate Dehydrogenase at 1.45 Å Resolution. Acta Crystallogr., Sect. F:Struct. Biol. Cryst. Commun. 2009, 65, 866–869. 10.1107/S1744309109032217. PMC2795587 19724119

[ref35] Buckle A. M.; Schreiber G.; Fersht A. R. Protein-Protein Recognition - Crystal Structural-Analysis of a Barnase Barstar Complex at 2.0-Angstrom Resolution. Biochemistry 1994, 33, 8878–8889. 10.1021/bi00196a004. 8043575

[ref36] Kussie P. H.; Gorina S.; Marechal V.; Elenbaas B.; Moreau J.; Levine A. J.; Pavletich N. P. Structure of the Mdm2 Oncoprotein Bound to the P53 Tumor Suppressor Transactivation Domain. Science 1996, 274, 948–953. 10.1126/science.274.5289.948. 8875929

[ref37] Weis W. I.; Kahn R.; Fourme R.; Drickamer K.; Hendrickson W. A. Structure of the Calcium-Dependent Lectin Domain from a Rat Mannose-Binding Protein Determined by Mad Phasing. Science 1991, 254, 1608–1615. 10.1126/science.1721241. 1721241

[ref38] Hakanpää J.; Linder M.; Popov A.; Schmidt A.; Rouvinen J. Hydrophobin Hfbii in Detail: Ultrahigh-Resolution Structure at 0.75 Å. Acta Crystallogr., Sect. D:Biol. Crystallogr. 2006, 62, 356–367. 10.1107/S0907444906000862. 16552136

[ref39] Wynne S. A.; Crowther R. A.; Leslie A. G. W. The Crystal Structure of the Human Hepatitis B Virus Capsid. Mol. Cell 1999, 3, 771–780. 10.1016/S1097-2765(01)80009-5. 10394365

[ref40] Rajagopal N.; Nangia S. Unique Structural Features of Claudin-5 and Claudin-15 Lead to Functionally Distinct Tight Junction Strand Architecture. Ann. N. Y. Acad. Sci. 2022, 1517, 225–233. 10.1111/nyas.14891. 36114674

[ref41] Sui H. X.; Han B. G.; Lee J. K.; Walian P.; Jap B. K. Structural Basis of Water-Specific Transport through the Aqp1 Water Channel. Nature 2001, 414, 872–878. 10.1038/414872a. 11780053

[ref42] Campaña M. B.; et al. The Ghrelin O-Acyltransferase Structure Reveals a Catalytic Channel for Transmembrane Hormone Acylation. J. Biol. Chem. 2019, 294, 14166–14174. 10.1074/jbc.AC119.009749. 31413115 PMC6768652

[ref43] Asthagiri D. N.; Beck T. L. Md Simulation of Water Using a Rigid Body Description Requires a Small Time Step to Ensure Equipartition. J. Chem. Theory Comput. 2024, 20, 368–374. 10.1021/acs.jctc.3c01153. 38156881

