# Supplementary material for: Extending the PARCH Scale: Assessing Hydropathy of Proteins across Multiple Water Models
Source: J Chem Inf Model. 2025 Mar 4;65(6):2999–3009. doi: 10.1021/acs.jcim.4c02415 (PMC11938274; doi:10.1021/acs.jcim.4c02415)
Supplement: Supplementary file 1 — ci4c02415_si_001.pdf [file ci4c02415_si_001.pdf]

Supporting Information

Extending the PARCH Scale: Assessing Hydropathy of Proteins Across Multiple Water Models

Xuyang Qin, Jingjing Ji, Somya Chakraborty, and Shikha Nangia*

Department of Biomedical and Chemical Engineering, Syracuse University, Syracuse, NY 13244, USA

*Address for correspondence:
Dr. Shikha Nangia
343 Link Hall
Department of Biomedical and Chemical Engineering
Syracuse University, Syracuse, NY 13244, USA
Phone (315) 443 0571 | Email: snangia@syr.edu
ORCID 0000-0003-1170-8461

Figure S1. The molecular structures of the TIP3P, TIP4P (and TIP4P-Ew), and TIP5P water models. 3
Figure S2. Radial distribution functions (RDF) of proteins simulated in TIP4P, TIP4-Ew, and TIP5P water models. 4
Figure S3. Autocorrelation curves. 6
Figure S4. Parch value profiles of amino acids in claudin-5 (CLD5) for four water models. 7
Figure S5. Parch value profiles of amino acids in melittin (MLT) for four water models. 8
Figure S6. Parch value profiles of amino acids in aquaporin (AQP1) for four water models. 9
Figure S7. Parch value profiles of amino acids in Ghrelin O-acyltransferase (hGOAT) for four water models. 10
Figure S8. Parch value profiles of amino acids in bacteriophage T4 lysozyme (LYM) for four water models. 11
Figure S9. Parch value profiles of amino acids in thymidylate synthase (TS) for four water models 12
Figure S10. Parch value profiles of amino acids in malate dehydrogenase (MDH) for four water models. 13
Figure S11. Parch value profiles of amino acids in barnase (BNS) for four water models. 14
Figure S12. Parch value profiles of amino acids in mannose-binding protein monomer (MBPm) for four water models. 15
Figure S13. Parch value profiles of amino acids in mannose-binding protein dimer (MBPd) for four water models. 16
Figure S14. Parch value profiles of amino acids in hepatitis B viral capsid monomer (HBVm) for four water models. 17
Figure S15. Parch value profiles of amino acids in hepatitis B viral capsid dimer (HBVd) for four water models. 18
Figure S16. Parch value profiles of amino acids in hydrophobin II (HP2) for four water models. 19
Figure S17. Parch value profiles of amino acids in mouse double minute 2 (MDM2) for four water models. 20
Figure S18. Statistical comparison (violin plots) of parch values for different proteins across four water models: TIP3P, TIP4P, TIP4P-Ew, and TIP5P. 21
Figure S19. Statistical comparison (violin plots) of parch values for different amino acids across four water models: TIP3P, TIP4P, TIP4P-Ew, and TIP5P. 22
Figure S20. The effect of position restraint force constant on protein structure during the parch annealing process. 23

Figure S1. The molecular structures of the TIP3P, TIP4P (and TIP4P-Ew), and TIP5P water models.

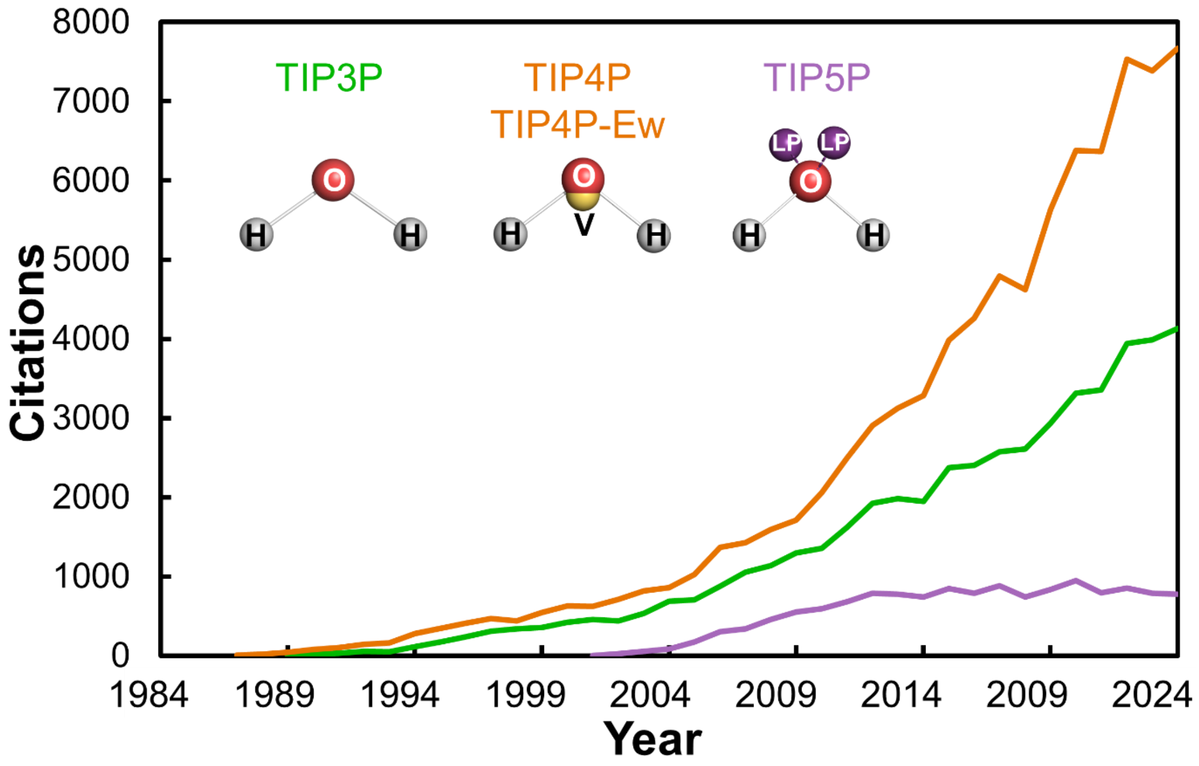

The oxygen (O), hydrogen (H), virtual site (V), and lone pairs of electrons (LP) are labeled for clarity. The graph depicts the annual citations for each model over the last three decades (1984 to 2024). The TIP4P and TIP4P-Ew models (orange) show a significant increase in usage in recent years, followed by TIP3P (green), while TIP5P (purple) has maintained relatively stable but lower levels of usage.

Figure S2. Radial distribution functions (RDF) of proteins simulated in TIP4P, TIP4-Ew, and TIP5P water models.

(A) The RDF of C_α of protein backbone and oxygen atom of TIP4P water

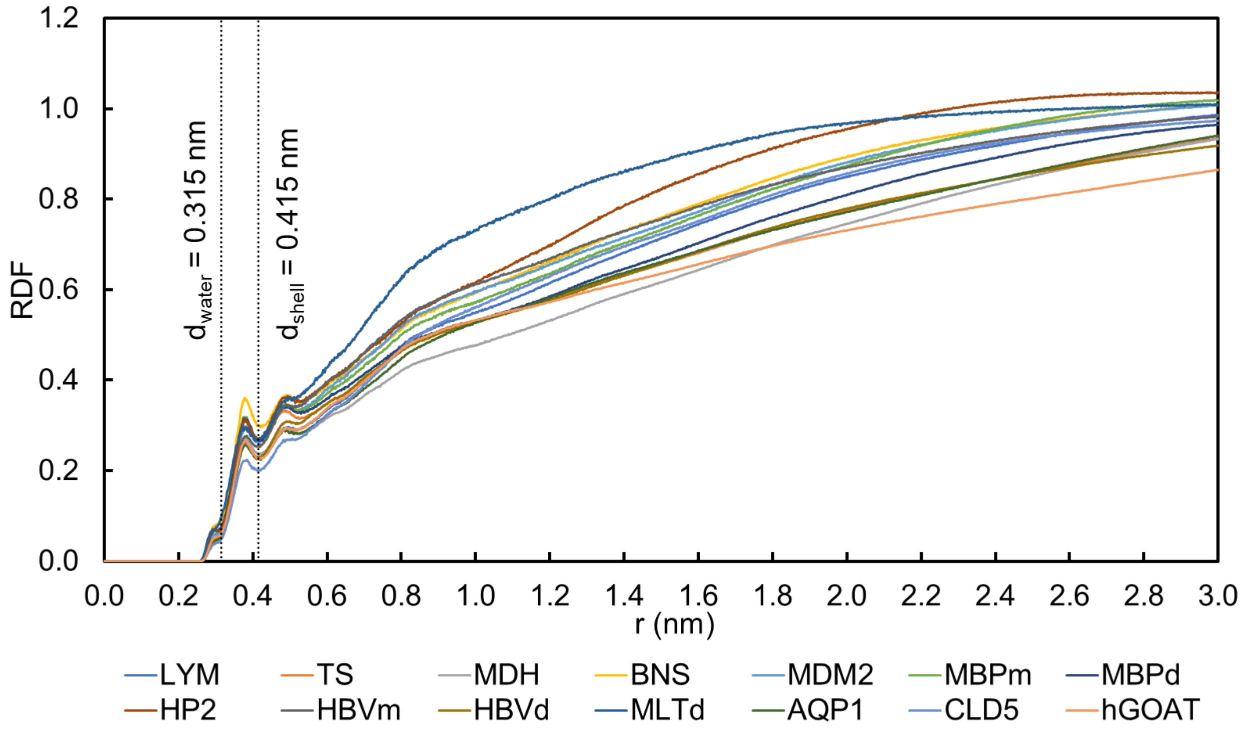

(B) The RDF of the backbone C_α of protein backbone and oxygen atom of TIP4P-Ew water

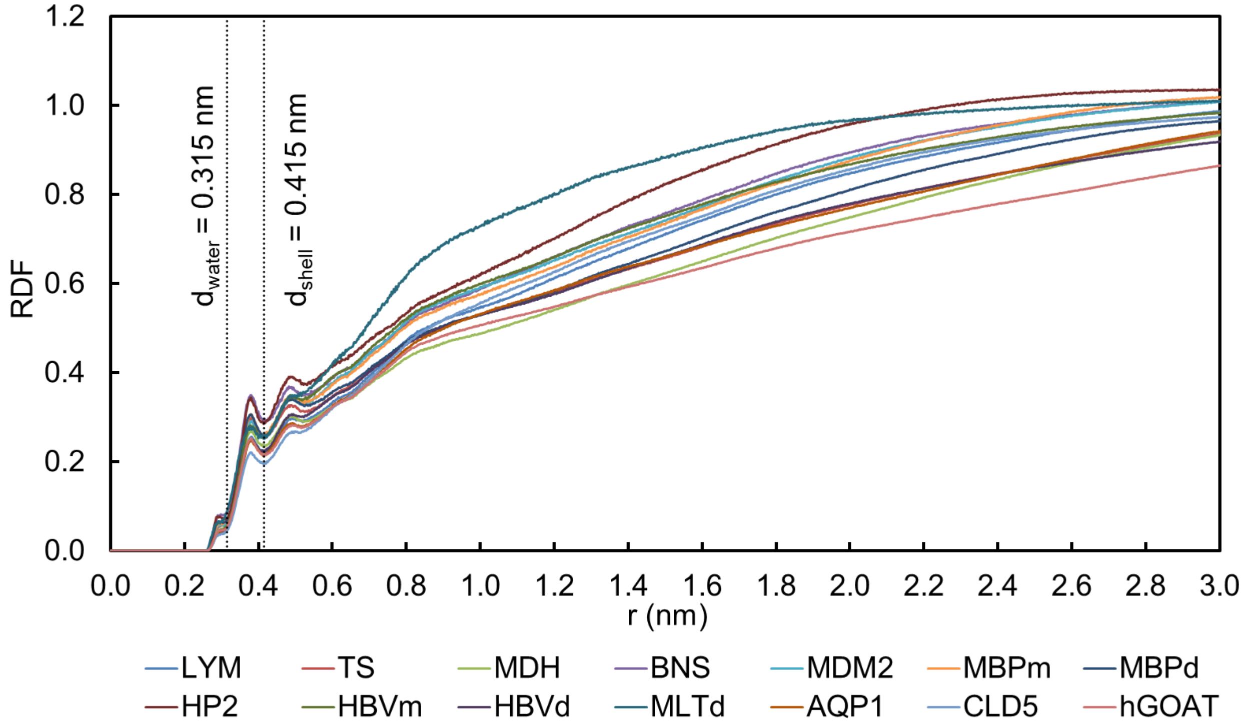

(C) The RDF of the backbone C_α of proteins and oxygen atom of TIP5P water

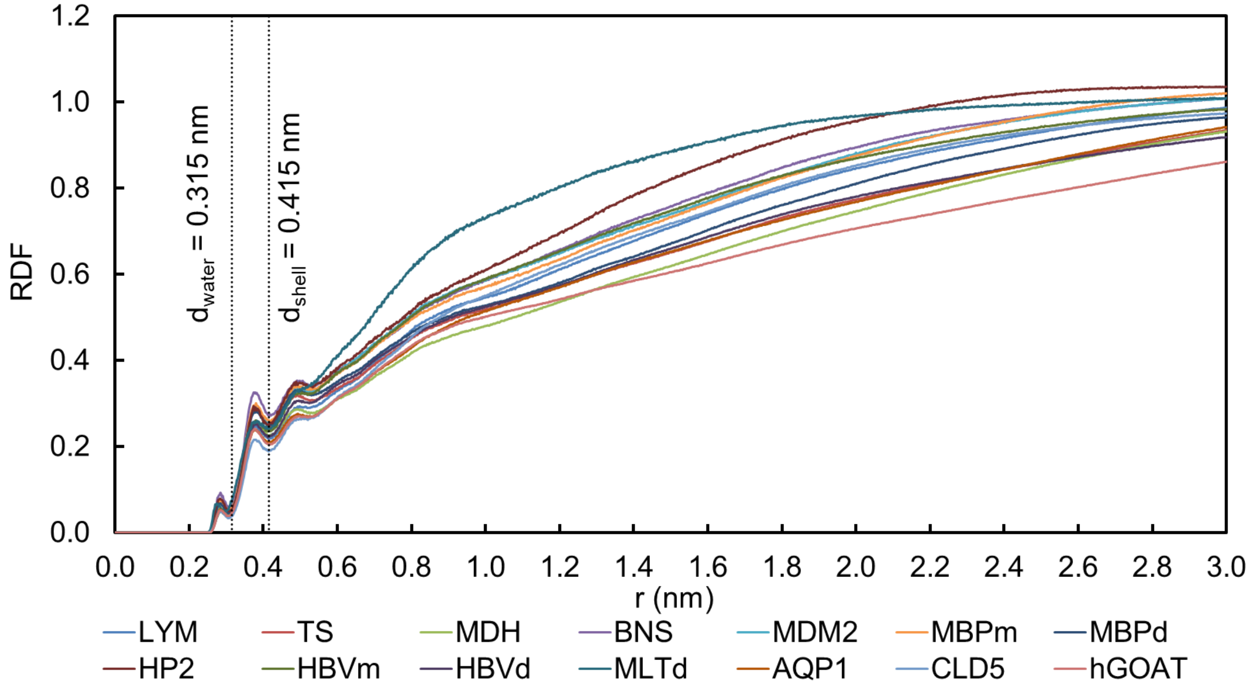

Figure S3. Autocorrelation curves.

(a) Single zwitterionic amino acids obtained using TIP3P water model.

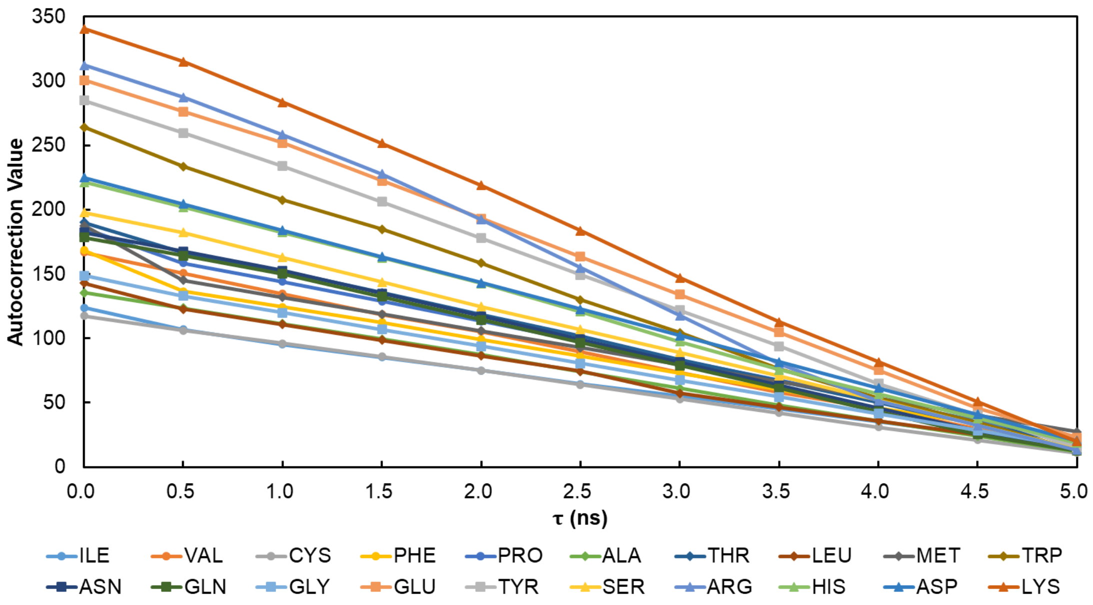

The autocorrelation curves for zwitterionic amino acids show long water retention times due to their charged N- and C-termini. In contrast, when the same amino acids are part of a protein backbone, they are held together by amide bonds, eliminating charged termini and having a more rapid water loss. As a result, the zwitterionic amino acid has higher PARCH values (Table 3) than when these residues are in protein environments (Figures 2B, 3C, 4B).

(b) MLT protein residues using TIP3P water model.

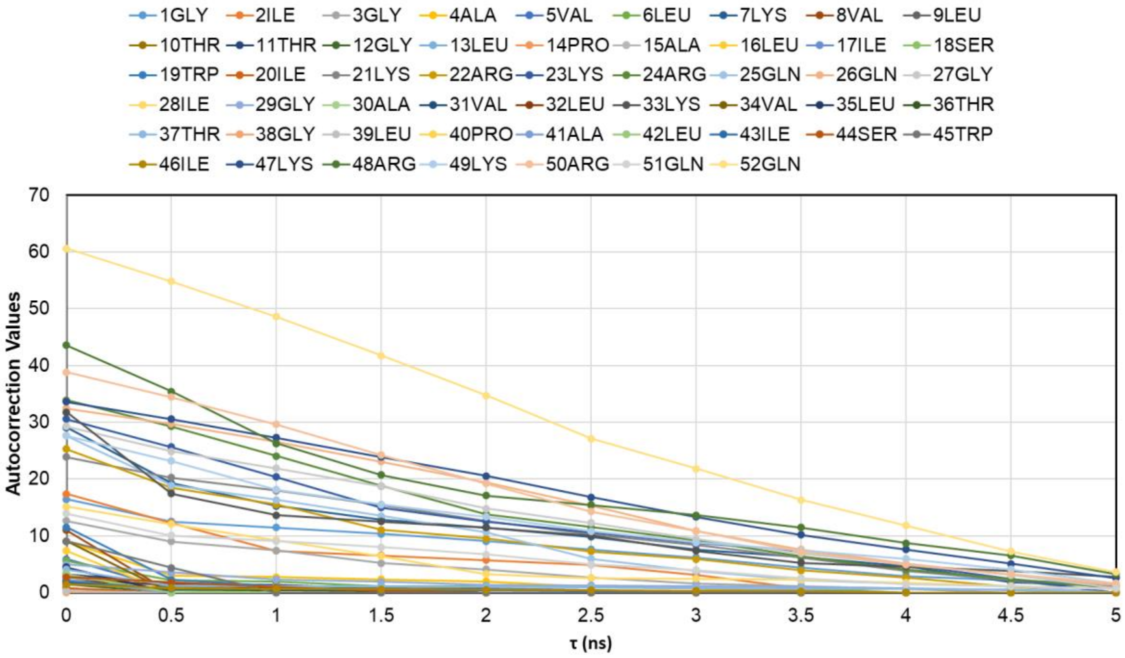

Figure S4. Parch value profiles of amino acids in claudin-5 (CLD5) for four water models.

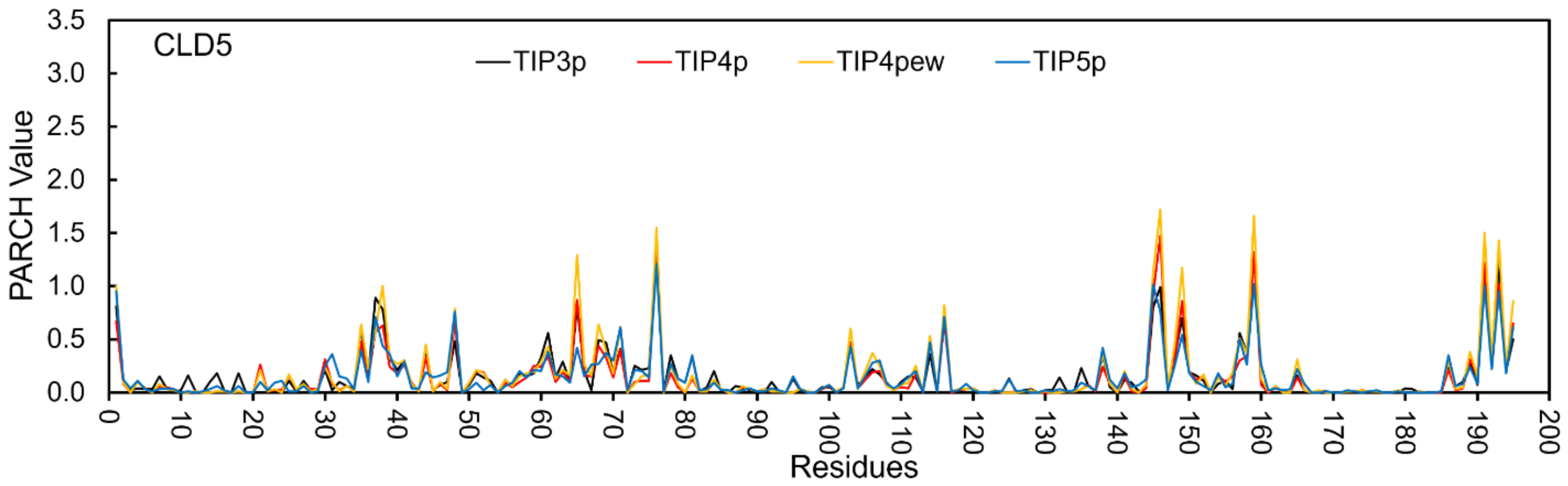

Figure S5. Parch value profiles of amino acids in melittin (MLT) for four water models.

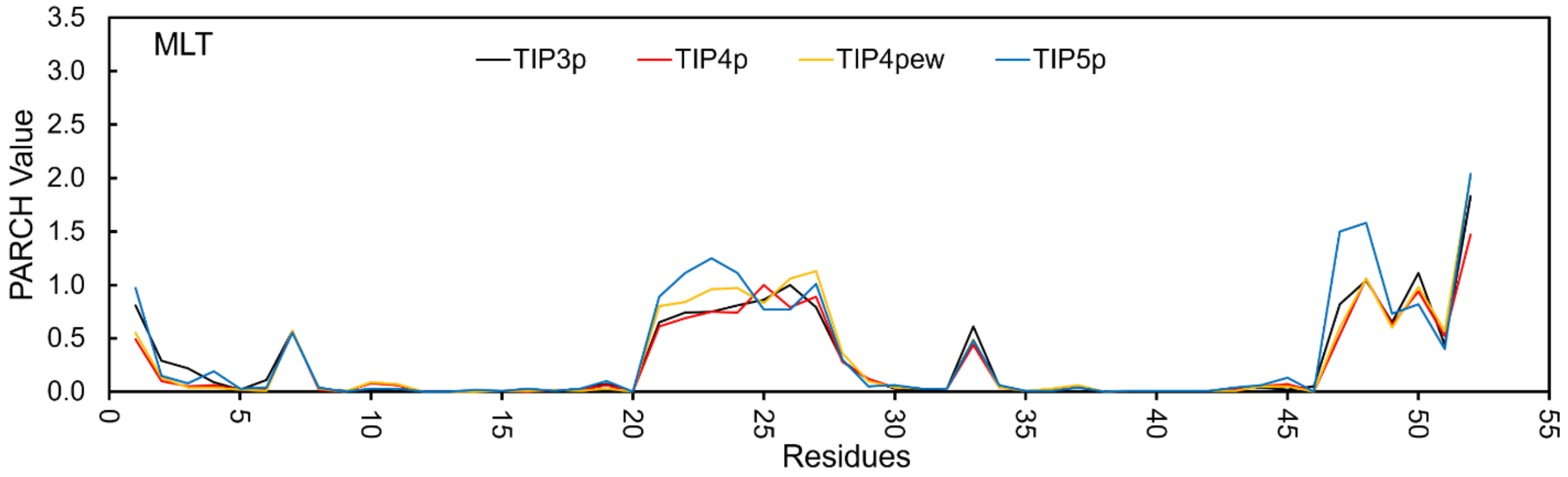

Figure S6. Parch value profiles of amino acids in aquaporin (AQP1) for four water models.

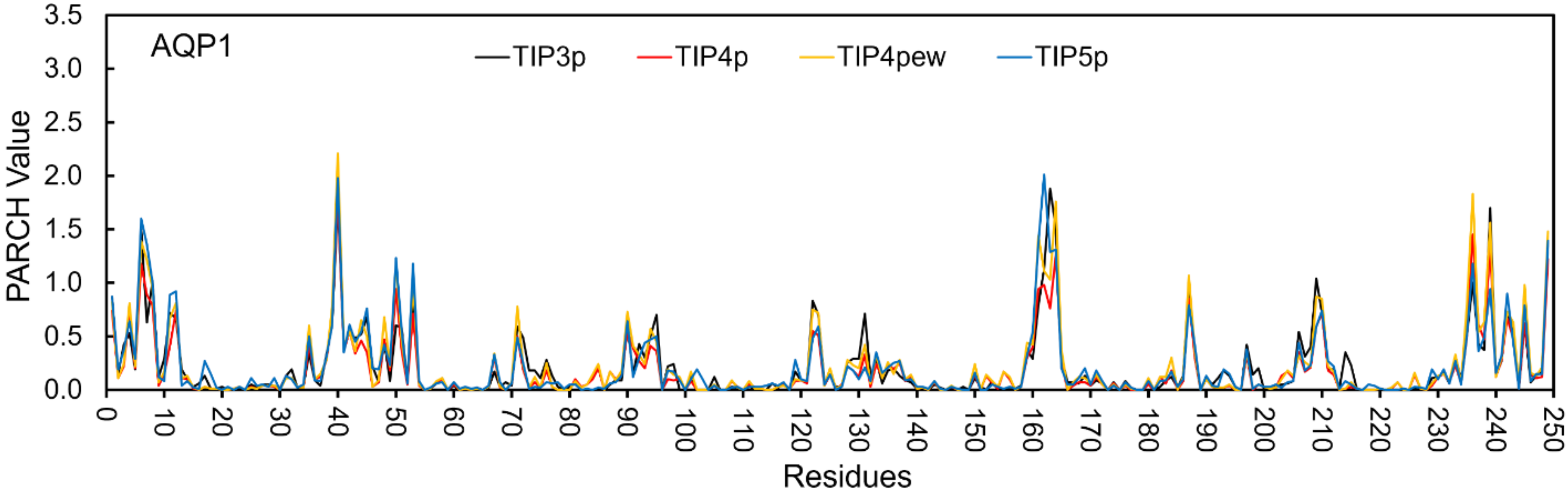

Figure S7. Parch value profiles of amino acids in Ghrelin O-acyltransferase (hGOAT) for four water models.

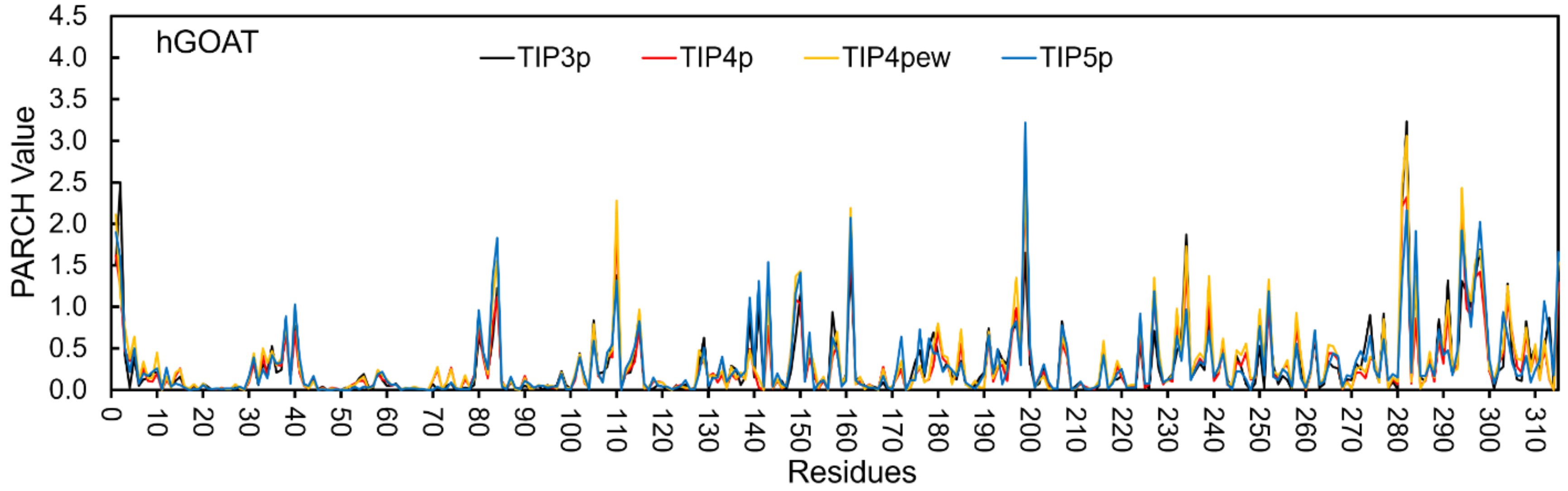

Figure S8. Parch value profiles of amino acids in bacteriophage T4 lysozyme (LYM) for four water models.

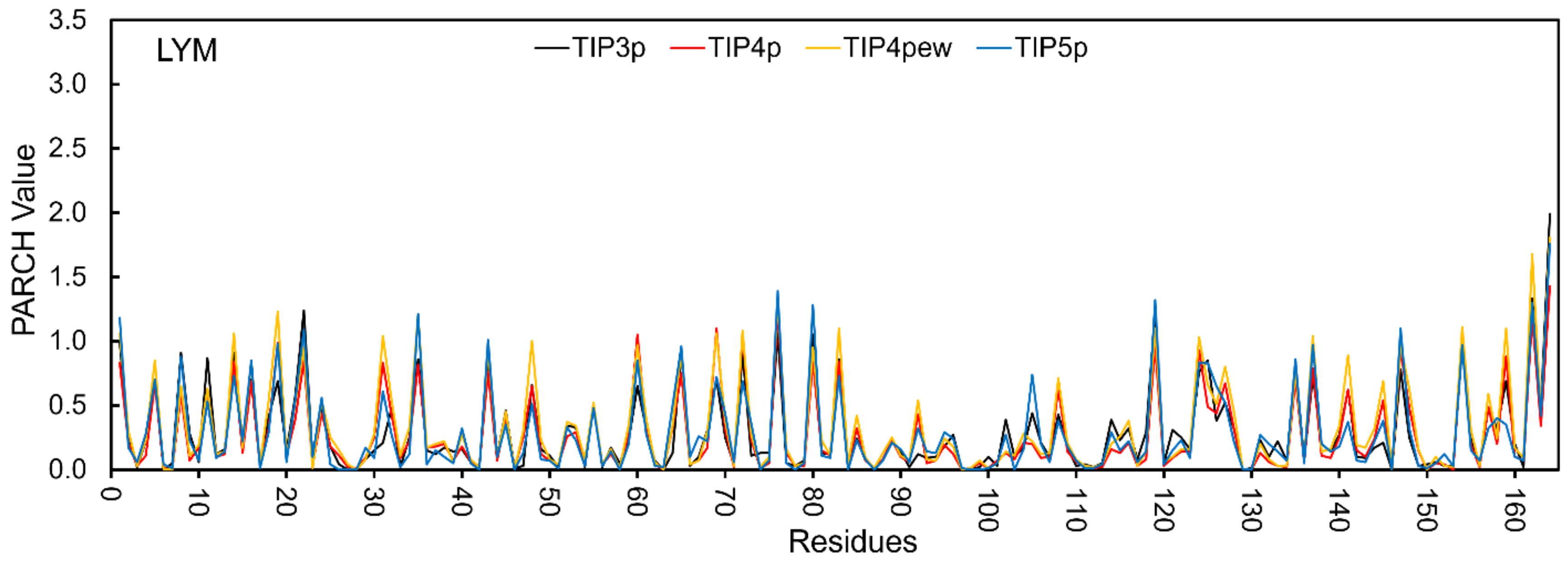

Figure S9. Parch value profiles of amino acids in thymidylate synthase (TS) for four water models

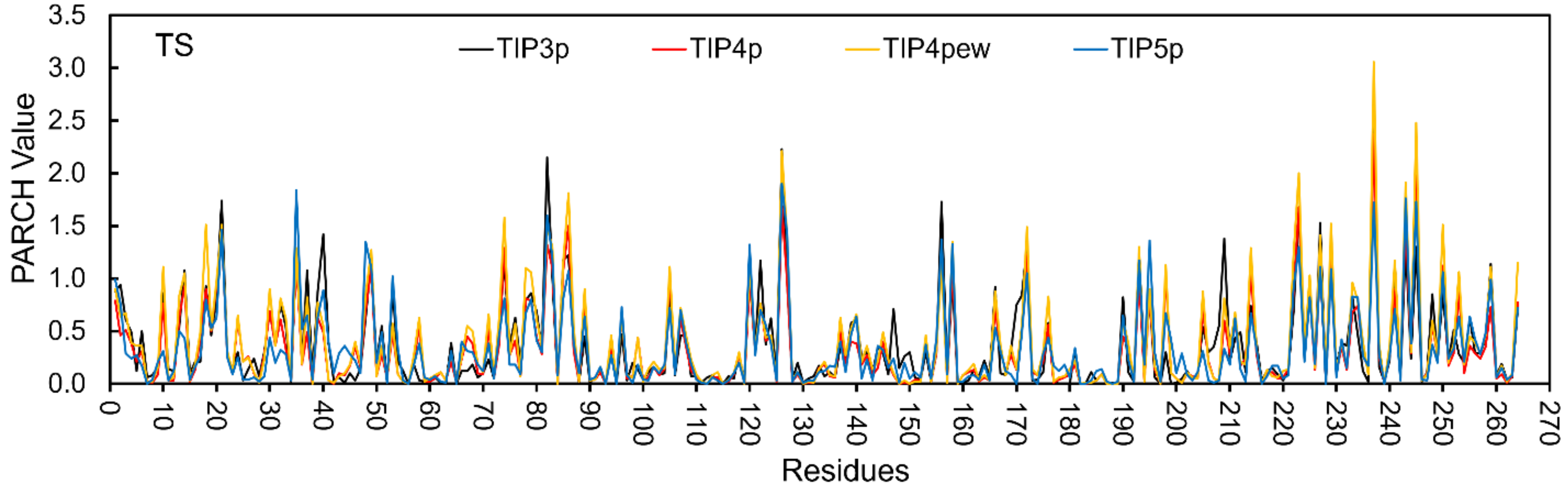

Figure S10. Parch value profiles of amino acids in malate dehydrogenase (MDH) for four water models.

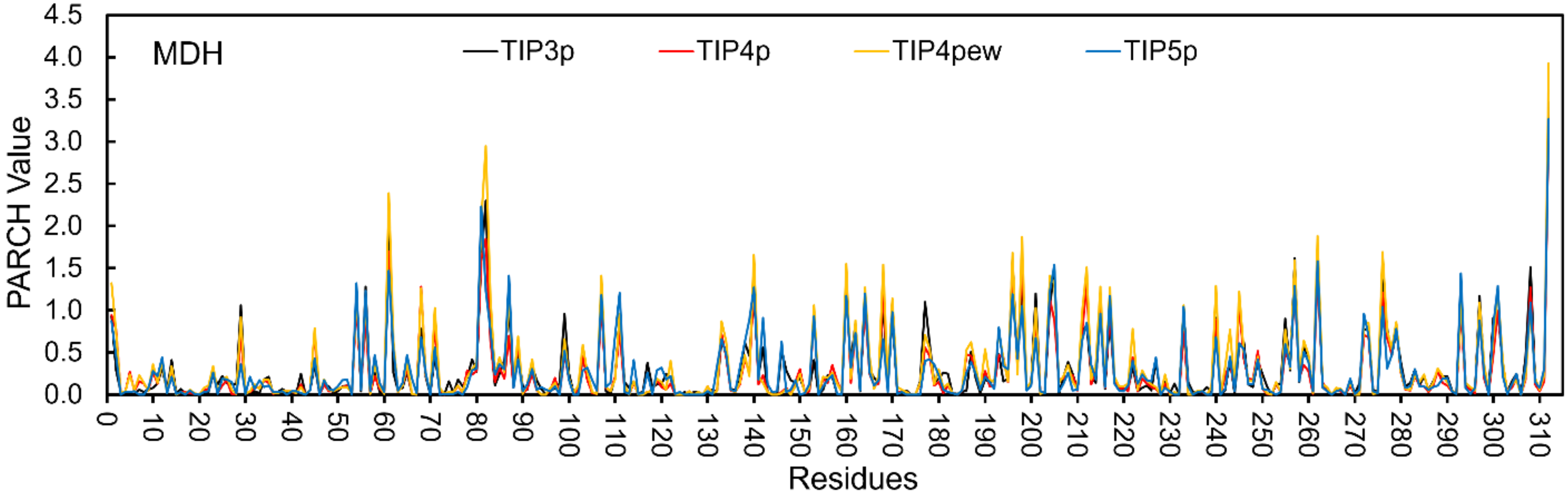

Figure S11. Parch value profiles of amino acids in barnase (BNS) for four water models.

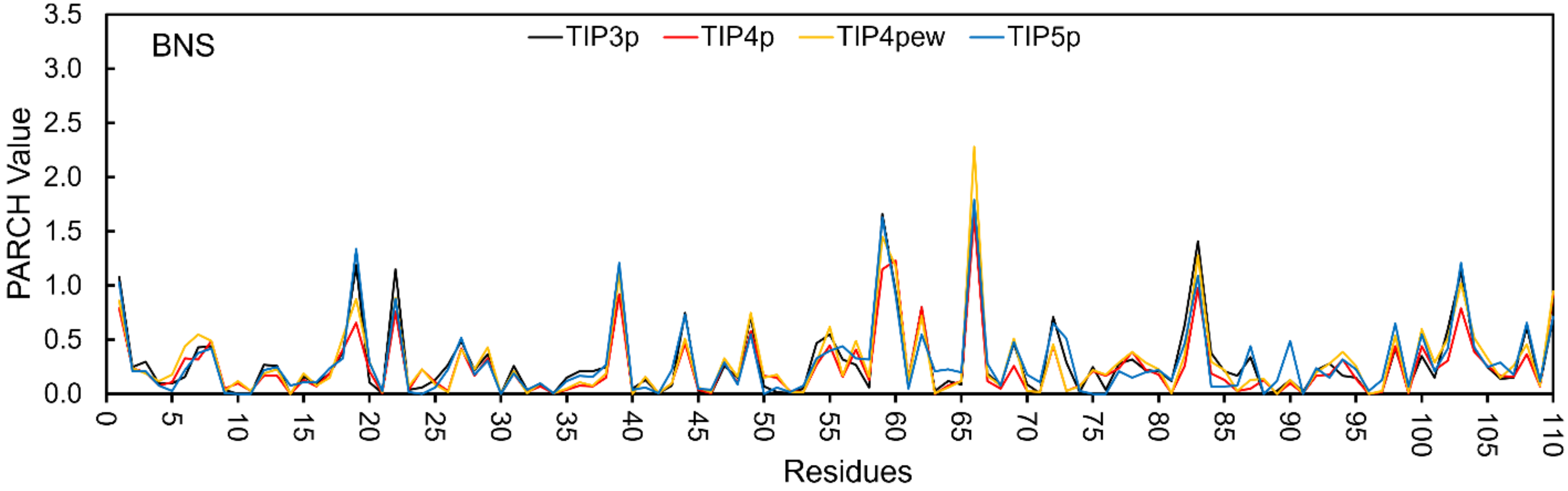

Figure S12. Parch value profiles of amino acids in mannose-binding protein monomer (MBPm) for four water models.

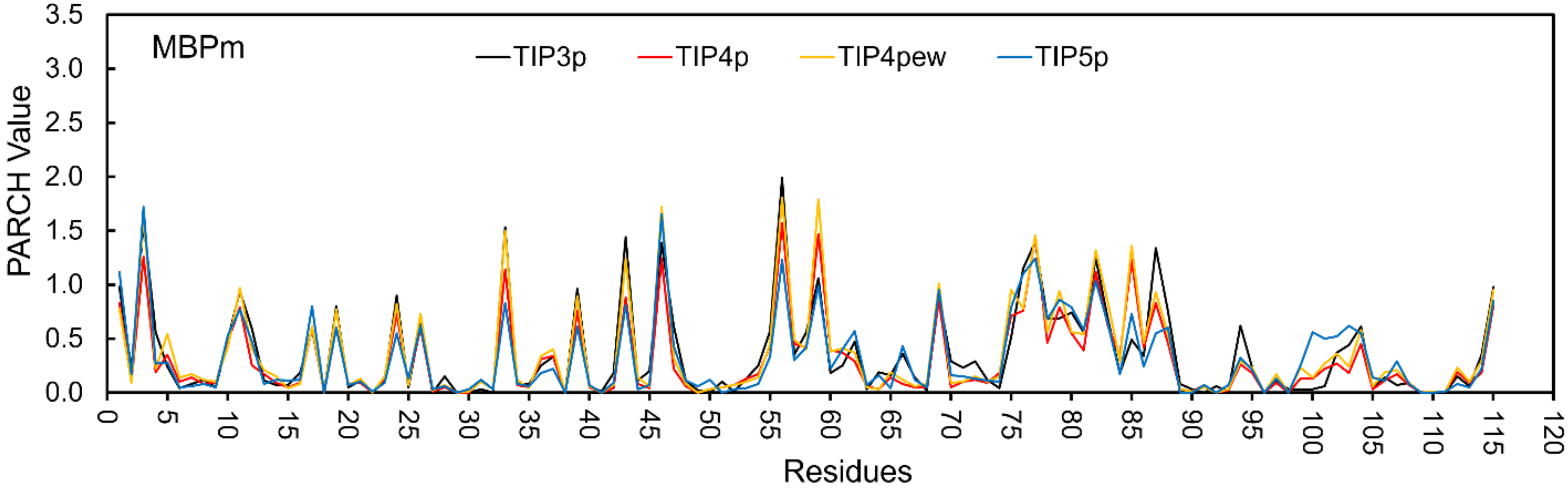

Figure S13. Parch value profiles of amino acids in mannose-binding protein dimer (MBPd) for four water models.

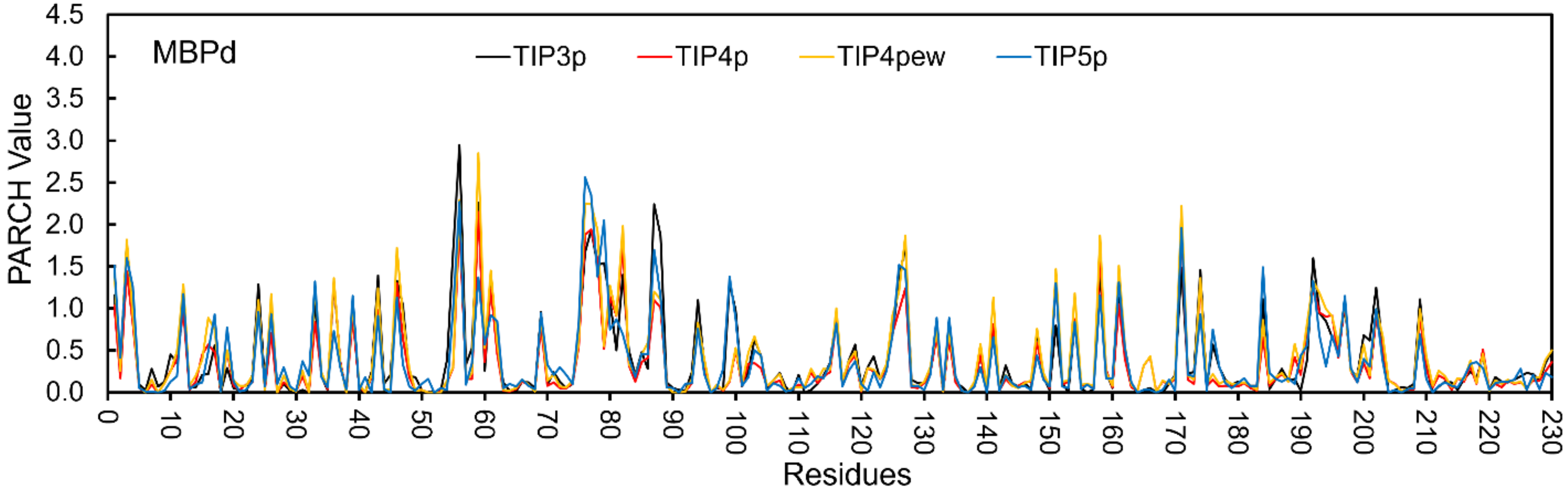

Note: The residues of two proteins are numbered in one sequence (115-230 is the second protein chain).

Figure S14. Parch value profiles of amino acids in hepatitis B viral capsid monomer (HBVm) for four water models.

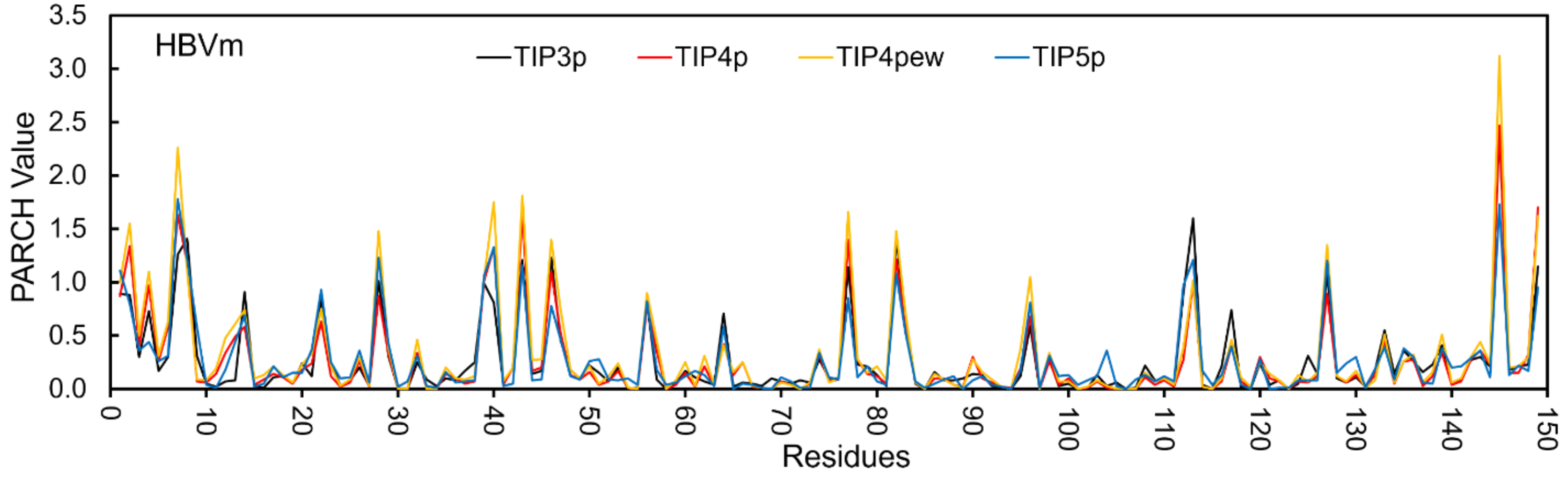

Figure S15. Parch value profiles of amino acids in hepatitis B viral capsid dimer (HBVd) for four water models.

Note: The residues of two proteins are numbered in one sequence (150-298 is the second protein chain).

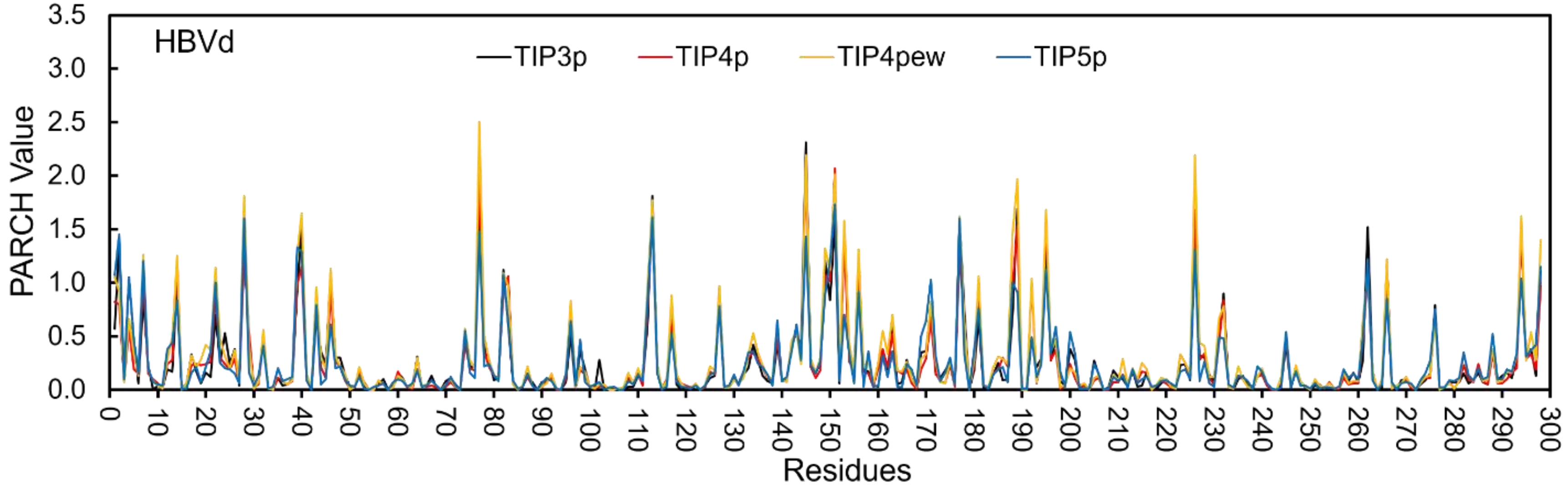

Figure S16. Parch value profiles of amino acids in hydrophobin II (HP2) for four water models.

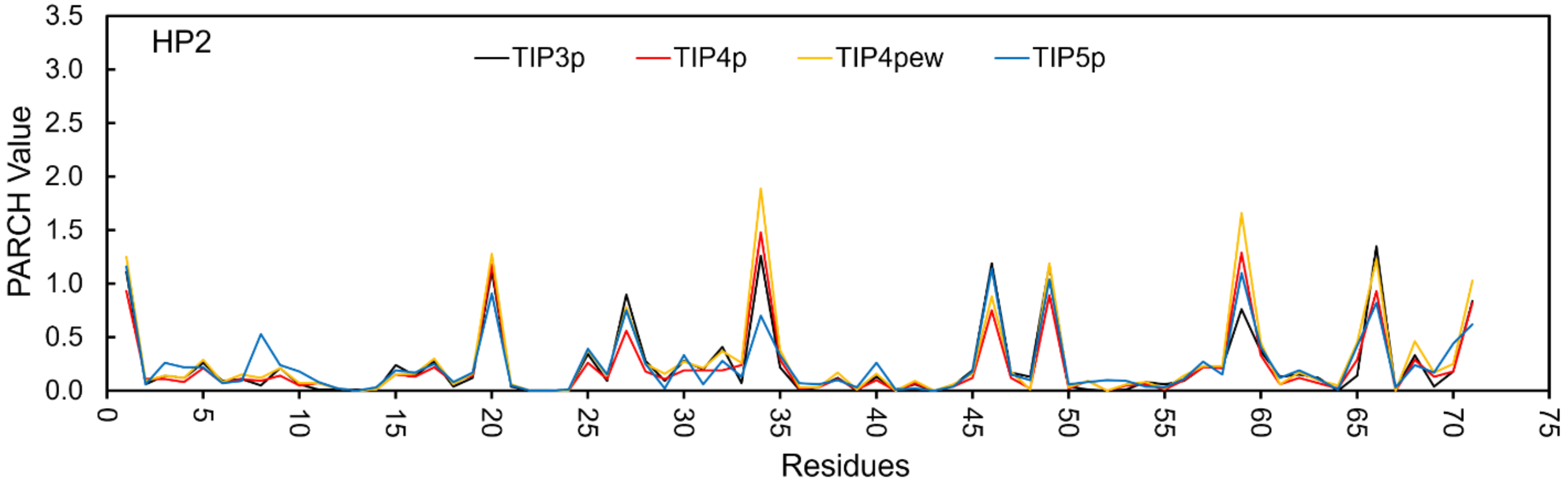

Figure S17. Parch value profiles of amino acids in mouse double minute 2 (MDM2) for four water models.

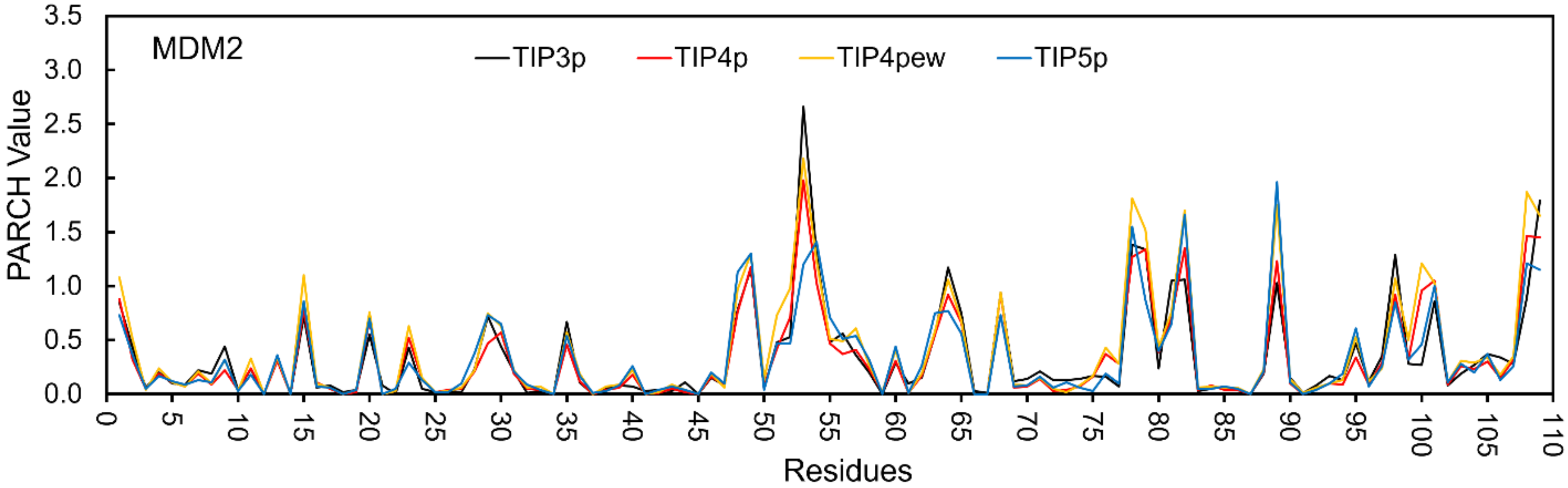

Figure S18. Statistical comparison (violin plots) of parch values for different proteins across four water models: TIP3P, TIP4P, TIP4P-Ew, and TIP5P.

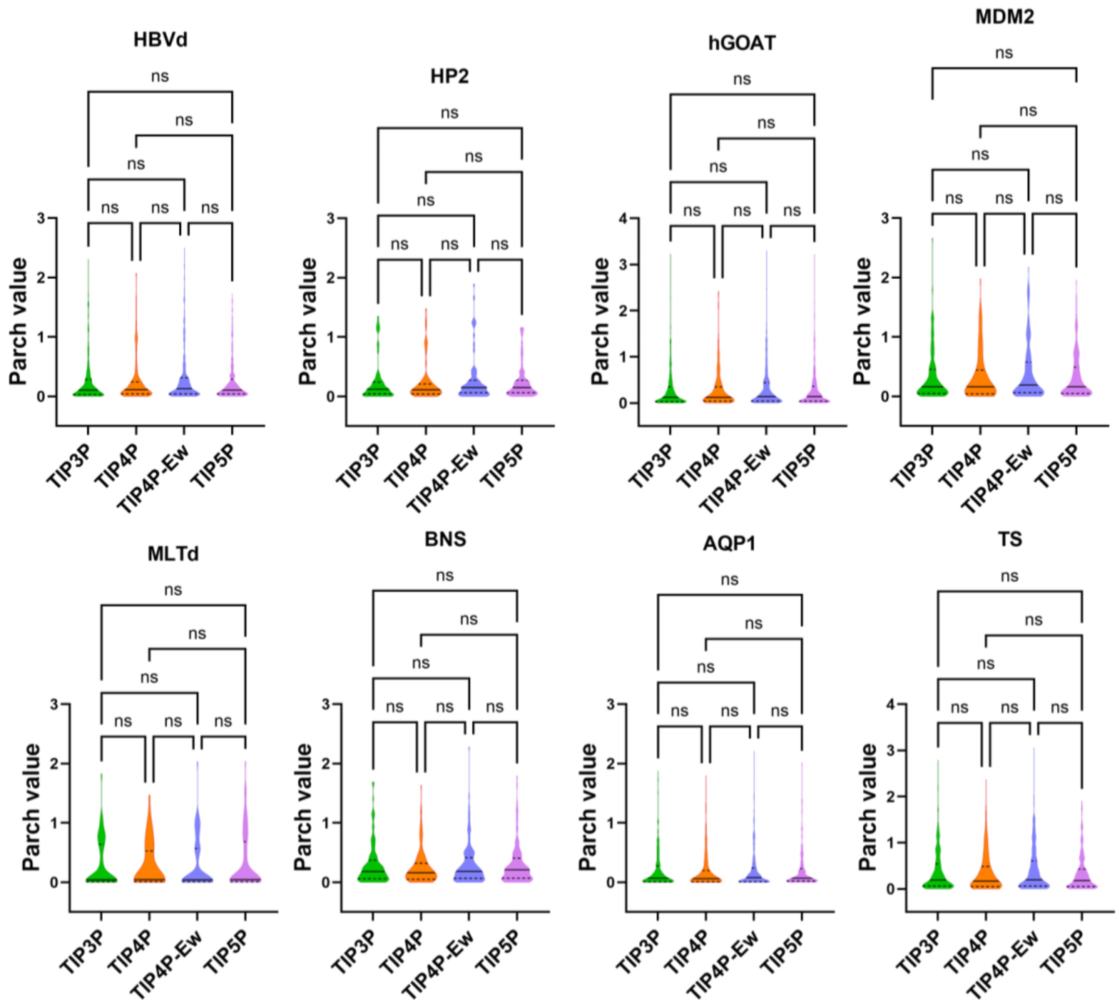

The plots illustrate the distribution of PV values, with colors representing the different water models. Statistical significance is indicated by labels: "ns" (not statistically significant, $p > 0.05$), while "*", "**", and "***" denote significant differences with $p \leq 0.05$, $p \leq 0.01$, and $p \leq 0.001$, respectively. The results highlight the absence of significant differences among water models for most amino acids based on the statistical tests applied.

Figure S19. Statistical comparison (violin plots) of parch values for different amino acids across four water models: TIP3P, TIP4P, TIP4P-Ew, and TIP5P.

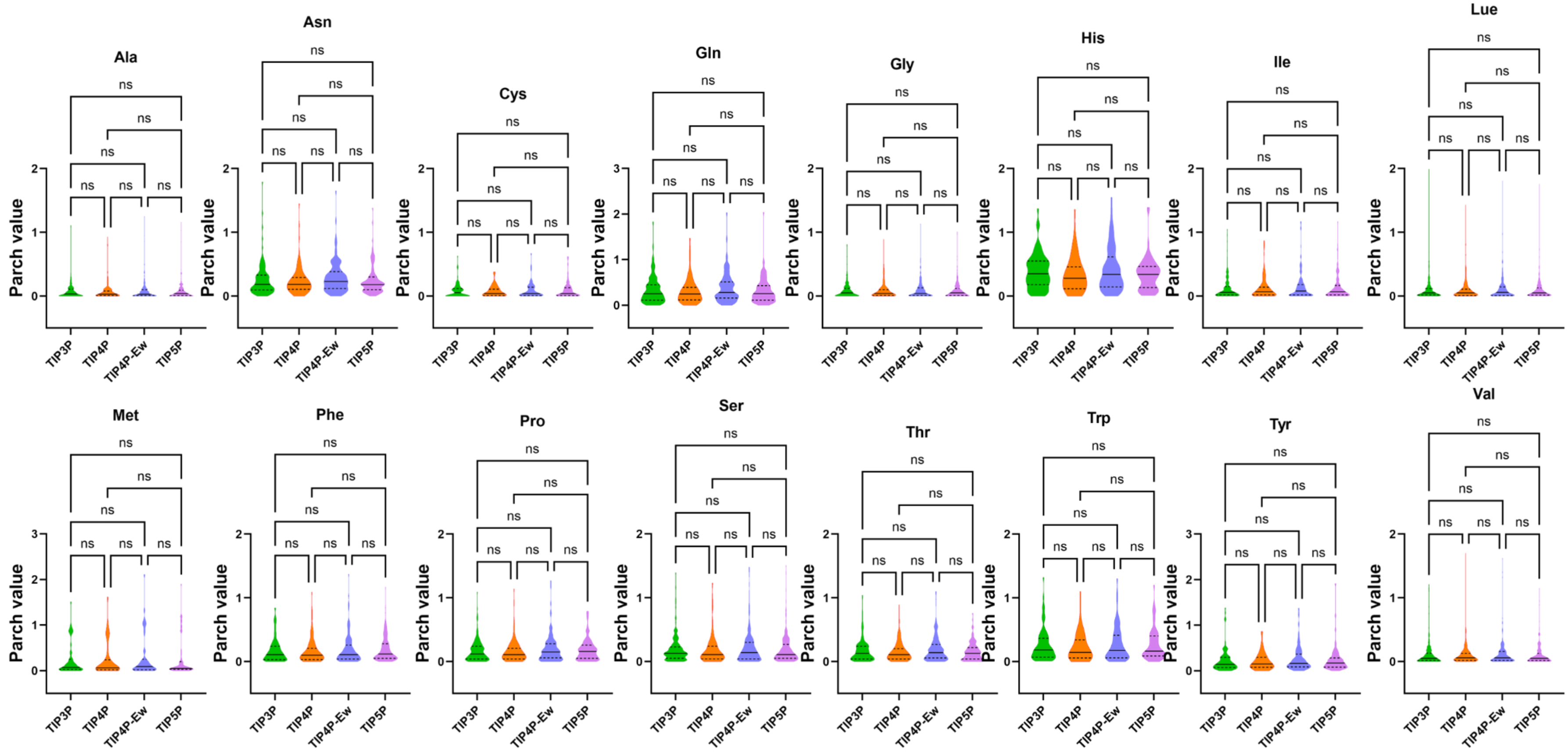

The plots illustrate the distribution of parch values, with colors representing the different water models. Statistical significance is indicated by labels: “ns” (not statistically significant, $p > 0.05$), while “*”, “**”, and “***” denote significant differences with $p \leq 0.05$, $p \leq 0.01$, and $p \leq 0.001$, respectively. The results highlight the absence of significant differences among water models for most amino acids based on the statistical tests applied.

Figure S20. The effect of position restraint force constant on protein structure during the parch annealing process.

RMSD was calculated for hGOAT and MLT, the largest and smallest proteins, respectively, among the fourteen proteins analyzed in this study.

(A) RMSD of hGOAT

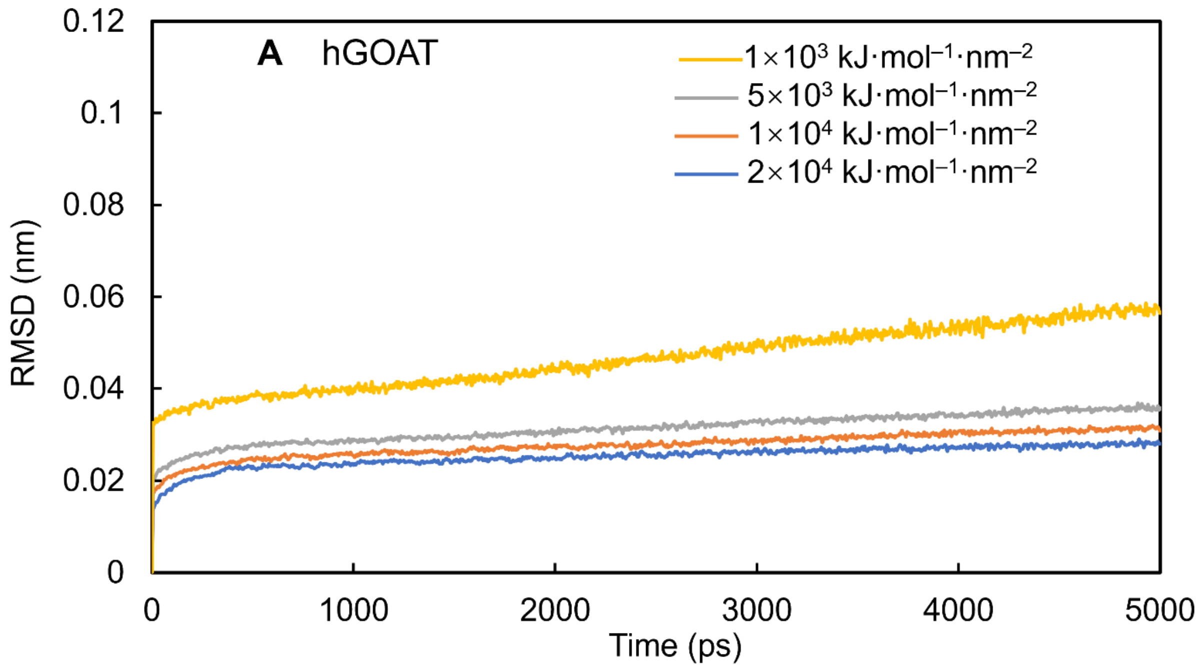

(B) RMSD of MLT

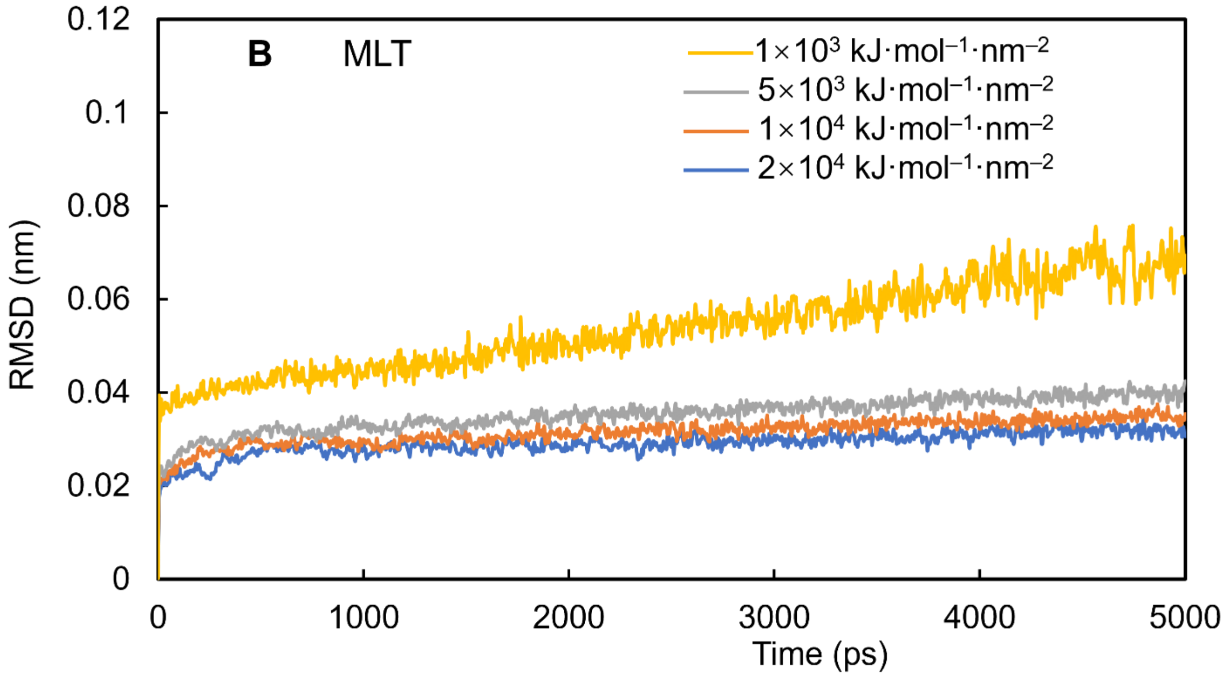

(C) Structural alignment of MLT at the initial and final states of annealing under varying position restraints: 1×10^3, 5×10^3, 10×10^3, and 20×10^3 kJ mol^{-1} nm^{-2}.

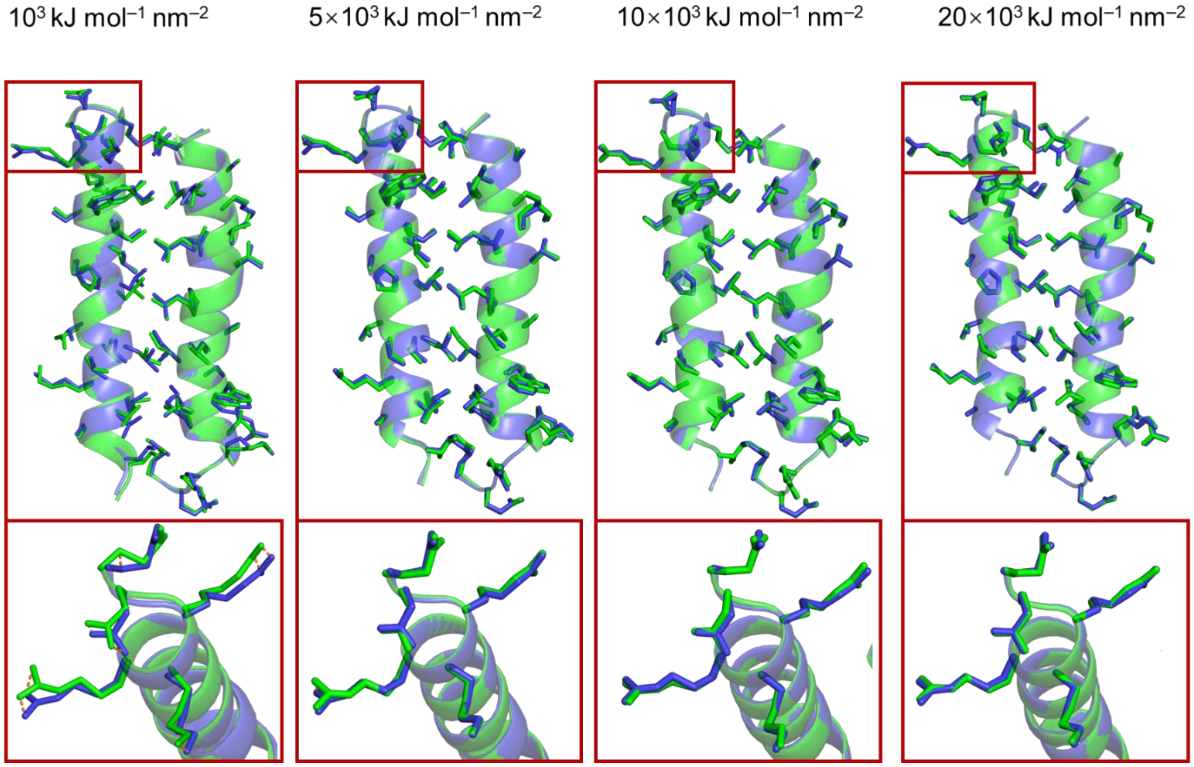

With a position restraint force constant of 10^3 kJ mol^{-1} nm^{-2}, the proteins exhibited RMSD values below 0.07 nm, a threshold typically considered indicative of system stability. However, some conformational changes were still noticeable, particularly in the side chains, shown in panel C of Figure S18. Since the parch scale accounts for structural conformations, such changes in the amino acid sidechain configurations can influence water evaporation during the annealing process and affect parch values.

We observed that a force constant of 10^3 kJ mol^{-1} nm^{-2} led to reduced protein stability at higher temperatures. Increasing the position restraint force constant to 5×10^3, 10×10^3, and 20×10^3 kJ mol^{-1} nm^{-2} resulted in lower RMSD values, indicating enhanced protein stability. However, the improvement in stability between 1×10^4 and 2×10^4 kJ mol^{-1} nm^{-2} was relatively minor. Based on these findings, a force constant of 1×10^4 kJ mol^{-1} nm^{-2} was chosen for this study.

.
